# Electro-optic modulation of coherent and incoherent mid-IR radiation in two-dimensional arrays

**DOI:** 10.1126/sciadv.aeg3189

**Published:** 2026-09-25

**Authors:** Jared Sisler, Phillippe Pearson, Michael Kelzenberg, Andrei Faraon, Harry A. Atwater

**Affiliations:** Thomas J. Watson Laboratories of Applied Physics, California Institute of Technology, Pasadena, CA 91125, USA.

## Abstract

Light in the mid-infrared (mid-IR) spans wavelengths from 3 to 8 micrometers and is important to many applications such as gas sensing and thermal imaging. Due to materials challenges, there is now a lack of mid-IR reconfigurable optical elements. Here, we present an electrically addressable metasurface for modulation of coherent and incoherent mid-IR radiation in two spatial dimensions. Our device achieves optical modulation due to the field-effect free-carrier depletion in a lightly doped (10^19^ per cubic centimeter) film of indium tin oxide coupled to a gap plasmon resonator. By addressing 32 individual elements across the metasurface, we first demonstrate tunable diffraction of coherently reflected mid-IR light. Next, we introduce a scalable perimeter-addressed driving scheme for tunable diffraction in two dimensions. Last, we demonstrate modulated emissivity with spatially reconfigurable two-dimensional patterns at elevated temperatures. This work advances the development of solid-state reflective beam-steering devices in the mid-IR and manipulation of thermally emitted incoherent radiation.

## INTRODUCTION

The mid-infrared (mid-IR) wavelength spectrum spans ∼3 to 8 μm. It is of scientific and technological interest because it contains the peak emission for most thermal bodies as well as characteristic absorption lines of many molecules. It is critical for gas detection (*1*), thermal energy harvesting (*2*), medical imaging (*3*), and military sensing (*4*). Despite widespread interest in the mid-IR, it now has a lack of optical components (i.e., sources, detectors, and modulators) compared with the near-infrared (near-IR) and visible. This gap in technology is primarily caused by limitations of available materials. For example, due to the lower energy mid-IR photons, detectors using photoabsorption are more difficult to design. Additionally, liquid crystals commonly used in spatial light modulators for visible and near-IR wavelengths are highly absorptive in the mid-IR, requiring molecular redesign for compatibility (*5*). Because of this, current techniques for active wavefront manipulation of coherent mid-IR radiation rely upon mechanically tunable mirrors. As an example, conventional digital micromirror devices consist of a two-dimensional (2D) array of microelectromechanical system–tunable mirrors that can impart binary amplitude profiles onto reflected mid-IR light for projection (*6*), spectroscopy (*7*), and adaptive beam-forming (*8*). While mirror-based wavefront shaping devices have high efficiency and amplitude contrast, their switching speed is limited to tens of kHz (*9*), phase modulation is difficult to achieve (*10*), and their pixel pitch is typically limited to a few to tens of microns. Optical phased arrays (OPAs) have been demonstrated in the mid-IR as a possible method to achieve dynamic beam steering (*11*), but their overall efficiency has been limited to a few percent and they are still restricted to modulation speeds in the tens of kHz due to the use of the thermo-optic effect for active phase shifting elements. Thus, there is now a need for devices capable of manipulating the wavefront of mid-IR light at high modulation rates (greater than MHz) with high efficiency and subwavelength spatial resolution.

Looking beyond the manipulation of coherent light, it has been shown that the radiation spectrum of a thermal body can be tailored by nanostructuring the material’s surface to introduce optical resonances (*12*–*14*). Additionally, the radiation pattern can be shaped to realize symmetric and asymmetric far-field diffraction (*15*–*17*), and various methods have been used to demonstrate dynamic modulation of spectral emissivity, such as electrical modulation of electron density coupled to a resonator (*18*–*21*). An interesting next step would be to demonstrate the tunable emissivity of a thermal emitter both in space and time. While there have been demonstrations of 2D arrays of electrically tunable thermal emitters, their modulation is based upon varying the local temperature (not the intrinsic emissivity) of each array element (*22*, *23*). It has also been shown that the directivity of thermal emission can be electrically switched, but, in this demonstration, the spatial emissivity profile across the device aperture was uniform (*24*). Thus, there is an opportunity for an active nanophotonic device capable of spatially and temporally modulating emissivity.

Here, we present an electrically addressable plasmonic metasurface for the dynamic modulation of coherent and incoherent mid-IR radiation across a 2D array, made possible by leveraging the fact that the emissivity of thermal bodies with temperatures between ∼50° to 700°C peaks in this wavelength range. First, we demonstrate the principle of our design by achieving tunable diffraction of mid-IR light in one dimension (Fig. 1A). Then, we introduce a straightforward modification of the electrical interconnects and optical antennas that allows tunable diffraction of mid-IR light in two dimensions and for any polarization of light (Fig. 1B). Last, by heating our device, we demonstrate spatially resolved dynamic emissivity modulation of thermally emitted light across our metasurface. This work presents a framework for designing active metasurfaces for the steering of coherent light and modulation of incoherent, thermally emitted light in the mid-IR.

**Fig. 1.**
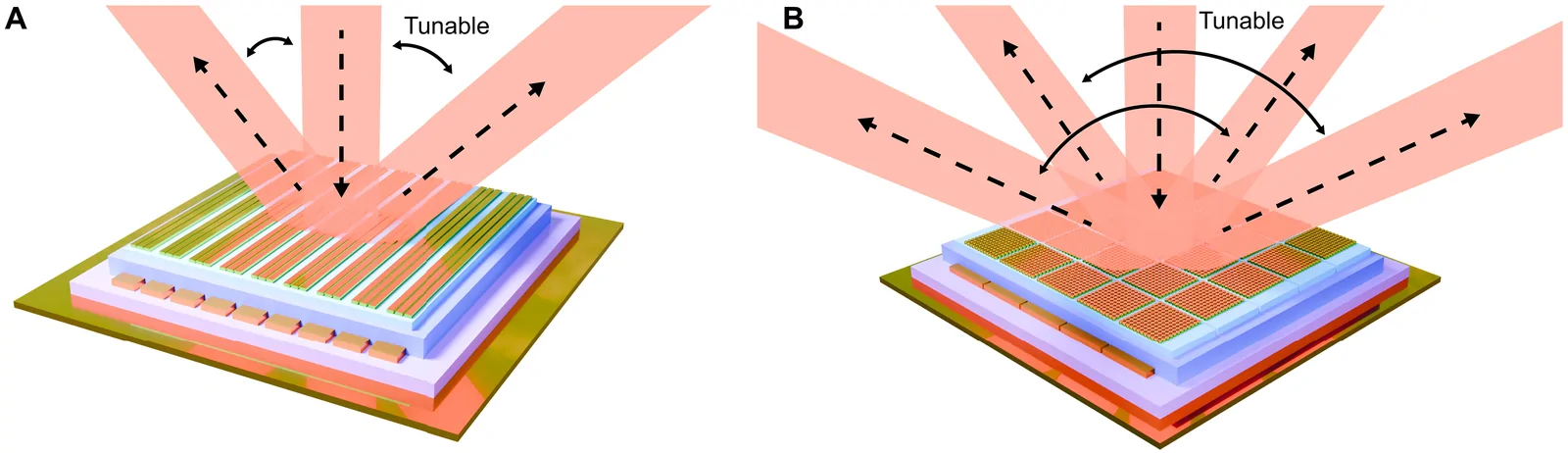
Active modulation of mid-IR radiation in two dimensions. (**A**) A 3D schematic of our active metasurface for the tunable diffraction in 1D of reflected coherent light in the mid-IR. (**B**) A 3D schematic of our active metasurface for the tunable diffraction in 2D of reflected coherent light in the mid-IR as well as the active modulation of thermally emitted light in 2D.

## RESULTS

### Design principles for an active plasmonic metasurface

Transparent conducting oxides (TCOs) are degenerately doped wide bandgap semiconductors that are typically transparent in the visible and near-IR. Some examples of common TCOs are indium tin oxide (ITO), aluminum-doped zinc oxide, gallium-doped zinc oxide, and cadmium oxide. The high density of charge carriers in TCOs is typically modeled as a “free electron gas,” which determines the optical response of the material. The dielectric function of a TCO can be described by the Drude-Lorentz that takes the form

$$\varepsilon \left(\omega \right)=\varepsilon_{\infty }-\frac{\omega_{p}^{2}}{\omega^{2}+i\text{ωΓ}}$$ (1)

where ε is the frequency-dependent complex permittivity, ε_∞_ is the high-frequency permittivity, and ω is the angular frequency of incoming light. The plasma frequency, ω_p_, and damping coefficient, Γ, are given by

$$\omega_{p}^{2}=\frac{Nq^{2}}{\varepsilon_{0}m_{e}^{*}}$$ (2)

and

$$\Gamma =\frac{q}{m_{e}^{*}\mu }$$ (3)

where *N* is the bulk carrier concentration, *q* is the elementary charge, ε_0_ is the permittivity of free space, $m_{e}^{*}$ is the effective mass of an electron, and μ is the electron mobility. An important feature of the Drude-Lorentz model is the existence of a frequency where the real part of the permittivity crosses zero, named the epsilon-near-zero (ENZ) point. At this frequency, the optical field becomes delocalized in the film, causing substantial absorption and many other interesting optical phenomena (*25*–*28*). The ENZ frequency is determined by four material parameters in the Drude-Lorentz model, *N*, ε_∞_, μ, and $m_{e}^{*}$. Among these, the carrier concentration, *N*, is the easiest to control experimentally and is often used to shift the ENZ frequency of a TCO film. ITO has been the most studied TCO material to date and is used as the active material in this work.

ITO (typically Sn-doped In_2_O_3_) can be deposited via many different techniques such as sputtering, evaporation, atomic layer deposition (ALD), and sol-gel processing (*29*). Of these deposition methods, radio frequency (RF) magnetron sputtering is the most common due to a favorable tradeoff between deposition rate, film quality and uniformity, film tunability, and capability for large area processing. A sputtering target containing a mix of Sn_2_O_3_ and In_2_O_3_ is typically used and sputtered in an environment of Ar and O_2_ (*30*). During deposition, the O_2_ flow rate and substrate temperature can be used to control the carrier concentration of the film (*31*). Postdeposition, annealing in various oxygen environments can also provide tunability, which we leverage here (*32*).

The free carriers in ITO are contributed from indium donors and oxygen vacancies. While the concentration of indium donors is intrinsic to the film, the density of oxygen vacancies can be changed after deposition. In most cases, the carrier concentration of ITO increases as oxygen vacancies increase. Thus, ITO films deposited with a low (high) oxygen flow rate tend to result in films with a high (low) oxygen vacancy concentration and thus a high (low) carrier concentration. This trend, however, only holds within a certain region of the parameter space dependent on other sputtering conditions such as substrate temperature, pressure, and power. Thus, it can be difficult to obtain a wide range of carrier concentrations by only controlling oxygen flow rate during deposition. Typically, achievable carrier concentrations are in the range of 1 × 10^20^ to 5 × 10^20^ cm^−3^ and correspond to ENZ frequencies in the near-IR. Postdeposition annealing in various oxygen environments can provide ENZ tuning over a wide spectral band (*32*), and Fig. 2A shows a visual schematic of how the concentration of oxygen vacancies in ITO can be manipulated by annealing in an oxygen-rich (air) versus an oxygen-poor environment (vacuum). The left-most film shows an as-deposited ITO film that consists of a fixed concentration of oxygen vacancies ($V_{O}^{\cdot \cdot }$) and oxygen interstitials ($O_{i}^{\prime \prime }$). During an anneal in air, there is a net movement of oxygen into the film which fills and removes oxygen vacancies, resulting in a decrease of the overall carrier concentration (Fig. 2A, top right). In contrast, annealing in vacuum will cause oxygen interstitials to exit the film, leaving behind oxygen vacancies, which increases the carrier concentration (Fig. 2A, bottom right).

**Fig. 2.**
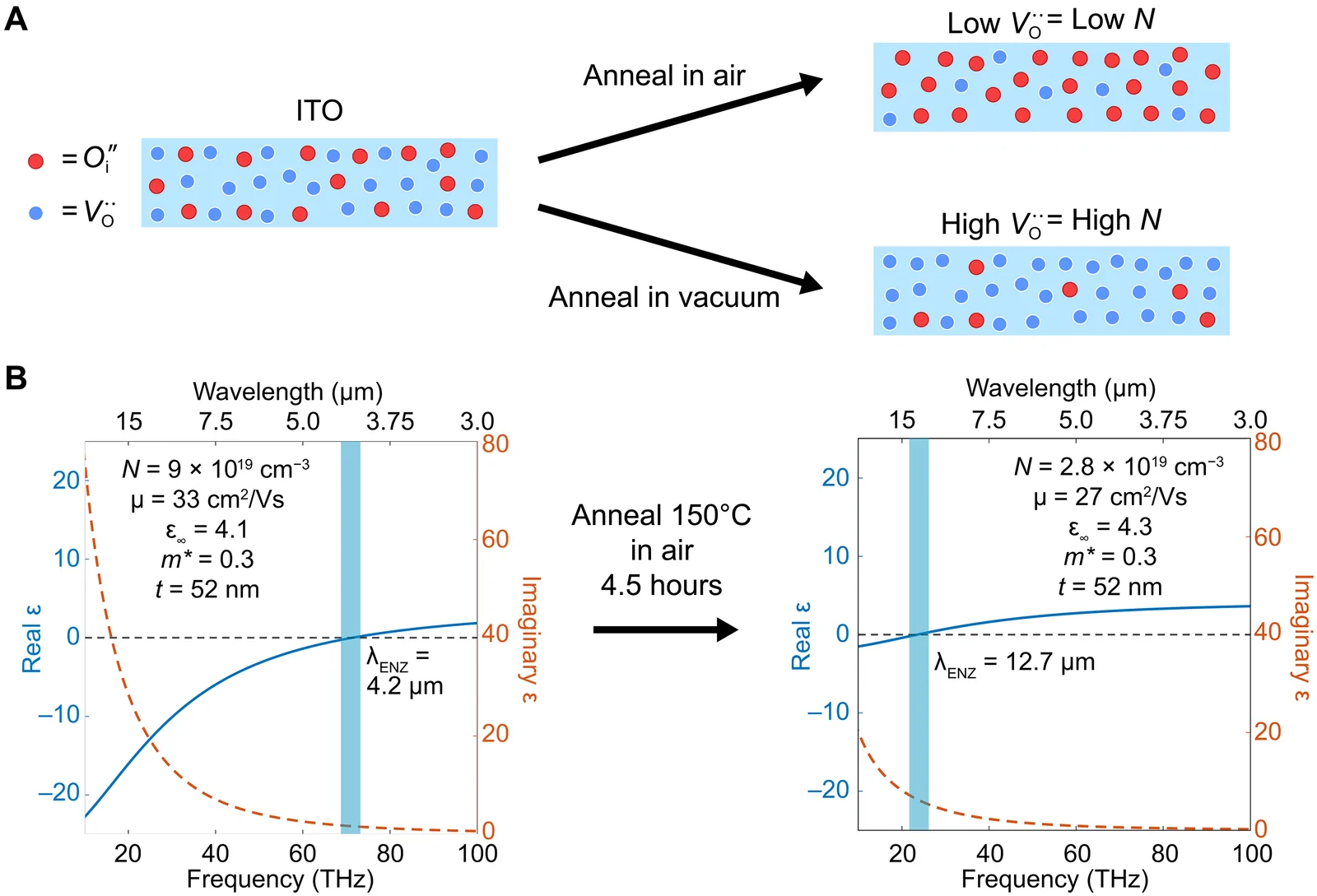
Engineering the properties of ITO. (**A**) ITO films can be modeled as a collection of oxygen interstitials, $O_{i}^{\prime \prime }$ (red circles), and oxygen vacancies, $V_{O}^{\cdot \cdot }$ (blue circles). The carrier concentration is proportional to the concentration of oxygen vacancies. After annealing in air (top right), the oxygen vacancy and carrier concentration is decreased. After annealing in vacuum (bottom right), the oxygen vacancy and carrier concentration is increased. (**B**) Experimentally measured permittivity, ε, of an ITO film directly after sputter deposition (left) and after a 4.5-hour 150°C anneal in air (right). The ENZ wavelength for each film is labeled and highlighted in blue.

As the ENZ frequency of ITO is typically in the near-IR, previous studies of ITO-based plasmonic metasurfaces for dynamic beam steering operate in this wavelength band (*33*–*36*). The device design in these demonstrations could be applied to other wavelength ranges such as the mid-IR and longwave-IR, but a substantial ENZ wavelength red shift of the ITO is required. Other materials such as n^+^-doped InAs have been explored for mid-IR devices (*21*), but the requirement for high-quality growth (often via molecular beam epitaxy) (*37*) and precise doping of InAs can make it difficult to integrate into complex optoelectronic devices. In this work, we develop a process that notably decreases the carrier concentration of ITO films following deposition. This approach allows us to precisely tune the ENZ wavelength and provides flexibility in the design of other layers in our device.

Figure 2B shows the real and imaginary permittivity components extracted by fitting to ellipsometric measurements of an as-deposited ITO film in our RF magnetron sputterer (left plot) and after a 4.5 hour anneal in air (right plot). These two measurements highlight our capability to shift the ENZ wavelength from ∼4 to 13 μm. By annealing at relatively low temperature (150°C), the diffusion of oxygen in and out of the film is very controlled such that it takes many hours or days to reach equilibrium. The final carrier concentration is highly dependent on the anneal time and allows us to precisely control the final properties of our ITO films. Thus, we have developed a controllable method to achieve a wide range of ENZ wavelengths in ITO films, widening the range of possible frequency bands for the operation of active optical devices.

### Active resonance modulation

Figure 3A presents a cross section of the active metasurface device. The uppermost Au layer consists of antennas whose period (Λ_ant_), width (*w*_ant_), and height (*h*_ant_) are 1400, 925, and 40 nm, respectively. These antennas sit on top of uniform layers of ITO (*h*_ITO_ = 50 nm) and Al_2_O_3_ ($h_{{\text{Al}}_{2}O_{3},2}$ = 160 nm). A layer of gold electrodes ($h_{\text{Au},1}$ = 80 nm) surrounded by Al_2_O_3_ enables electrical tuning of a *p*-polarized gap plasmon resonance hosted between the antennas and electrodes. Each electrode spans three antenna periods, with voltages applied between the bottom Au electrodes and top ITO layer to modify the carrier concentration of the ITO. This results in a local modulation of the reflection coefficient above each electrode, as schematically depicted in Fig. 3A. Throughout the rest of the text, we refer to the individual uppermost gold elements as antennas and the wider, electrically addressable elements beneath them as electrodes. In this device, only the electrodes are electrically driven, while the antennas are needed to form the plasmonic resonator. We chose to group antennas in this way to, first, reduce the complexity of the driving electronics such that we require only 32 electrical channels to cover a 400 μm-wide aperture. Second, the pitch of the addressable electrodes (Λ_electrode_ = 3 × Λ_ant_ = 4200 nm) remains subwavelength to suppress unwanted diffraction orders and allow for the steering of light over a wide field of view. Third, grouping antennas will minimize cross-talk between adjacent electrodes (see section S1 for visualizations of the DC and optical fields in the electrodes). This is an important advantage of our device design compared with others such as liquid crystal spatial light modulators, which inherently suffer from pixel-to-pixel cross-talk. The electrodes are separated from a back gold film ($h_{\text{Au},2}$ = 80 nm) with a layer of uniform Al_2_O_3_ ($h_{{\text{Al}}_{2}O_{3},1}$ = 200 nm) to prevent radiation from being transmitted through the device. The metasurface is wirebonded to an interposer printed circuit board (PCB), which interfaces with a set of custom-built programmable PCBs capable of applying voltages between −30 and 30 V to 32 individual channels, each corresponding to a gold electrode. Additionally, we apply a constant voltage offset of 30 V (*V*_offset_ in Fig. 3A) to allow our ±30 V voltage source to drive each antenna into depletion with a range of 0 to −60 V because our device is designed for maximum modulation when the ITO layer is depleted of carriers (see section S2). The electrodes corresponding to the 32 independent voltage channels span a 134.4 μm linear dimension, and this set of 32 is repeated three times to form the full device, which measures ∼400 μm × 400 μm with one extruded dimension. In Fig. 3B, a scanning electron micrograph shows the antennas patterned above the addressable electrodes, and the inset provides a magnified view of a single electrode. Section S3 provides additional optical and scanning electron microscopy images of our active metasurfaces.

**Fig. 3.**
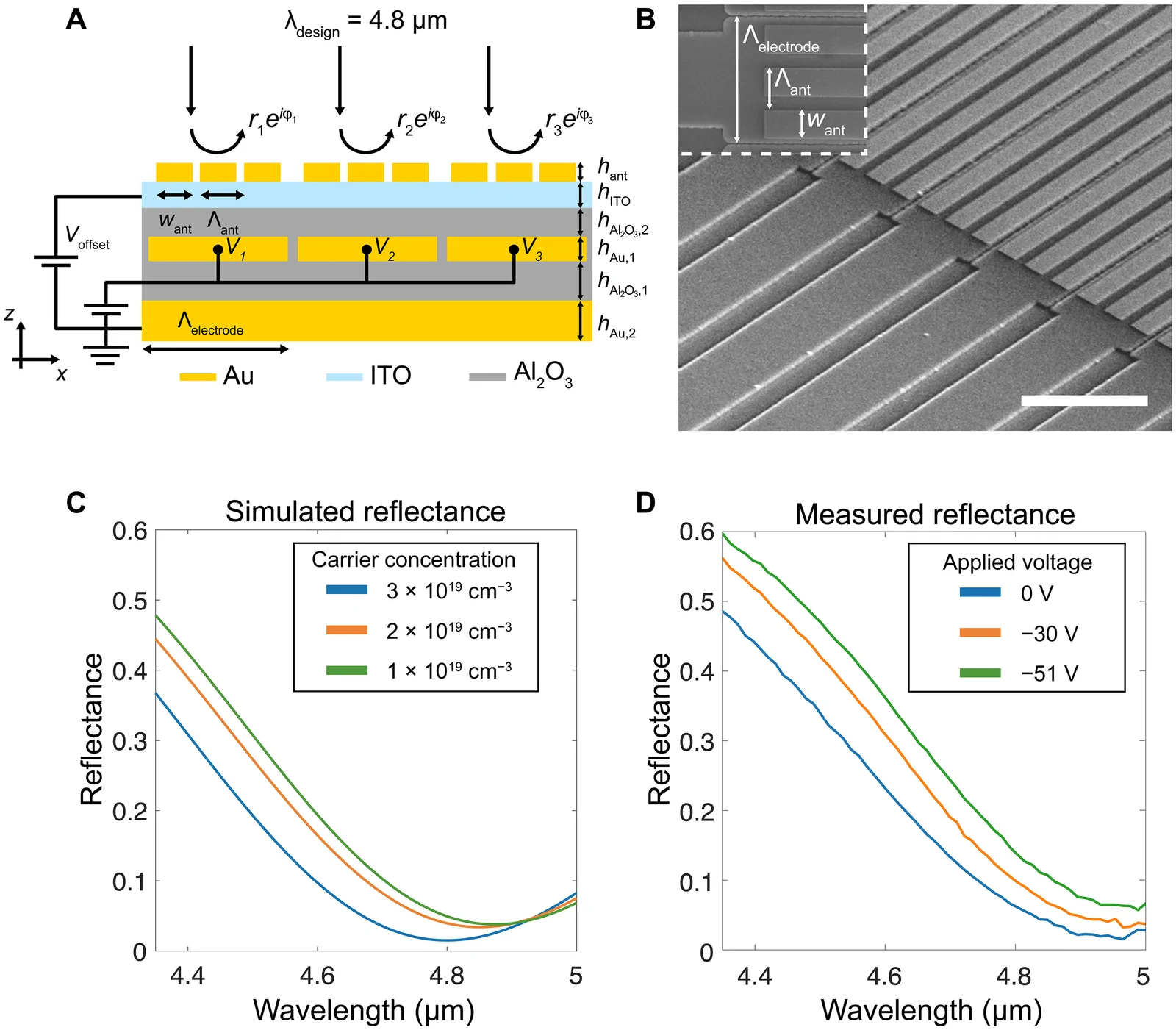
1D active metasurface platform. (**A**) Cross-section of the device showing the local modulation of the reflection coefficient with applied voltage. (**B**) Scanning electron micrographs of the fabricated metasurface. The inset shows a closer view of one set of three antennas. Scale bar for larger image, 5 μm. Labeled widths in inset: *w*_ant_ = 910 nm, Λ_ant_ = 1.4 μm, and Λ_electrode_ = 4.2 μm. (**C**) Simulated reflectance of the metasurface at normal incidence for *p*-polarized illumination for different assumed carrier concentrations. (**D**) Measured *p*-polarized reflectance when applying three uniform voltages across the ITO and electrodes.

We begin by characterizing the electrostatic tuning of the metasurface resonance. When a negative voltage is applied to the addressable elements, the ITO becomes depleted of charge carriers near its interface with the top Al_2_O_3_ layer. This, in turn, modulates the gap plasmon resonance hosted between the gold antennas and the addressable gold electrodes below, enabling local control of scattered light. We simulate the reflectance of this structure with a finite-difference time-domain (FDTD) solver (Lumerical Inc.) under *p*-polarized illumination at normal incidence between λ = 4.35 and 5 μm. Figure 3C shows the simulated reflectance spectra for a series of assumed carrier concentrations in a 10 nm region of ITO to approximate a depletion layer. As discussed above, the optical properties of ITO are captured by the Drude-Lorentz model and, based on electrostatic simulations and analytical calculations, we assume that the bottom 10 nm of the ITO layer (adjacent to its interface with Al_2_O_3_) is modulated when in depletion. Justification of this thickness is provided in section S2. Because the field of the gap plasmon is highly concentrated in this region, the simulated spectrum shifts as the carrier concentration is reduced. This is in contrast to prior work with ITO-based active metasurfaces where the carrier concentrations are typically much larger and only a ∼1 nm layer is modulated with carrier accumulation. Because our carrier concentration is an order of magnitude lower, the volume of the depletion layer is roughly an order of magnitude larger and results in strong modulation.

The measured reflectance spectra also shift appreciably with applied negative voltage (Fig. 3D). A detailed description of the measurement setup is given in section S4. At 0 V, the resonance is centered near λ = 4.95 μm, which is red shifted relative to the design wavelength of 4.8 μm. We are limited to measuring between λ = 4.35 and 5 μm due to the tuning range of our laser (MIRcat, Daylight Solutions Inc.) and the onset of a strong absorption line of CO_2_ near 4.2 μm, which strongly limits the detected reflected power. The resonant frequency of the fabricated device likely differs from that of the simulated one because of differences in the carrier concentration of the ITO from what is assumed in simulation and measured before depositing the gold antennas. Despite this, on resonance, the reflectance increases from ∼2 to 9% when applying −51 V to the device, which is in agreement with the trend from simulation.

### Active beam steering in one dimension

In the previous section, we studied the ability of our metasurface to modulate incident light uniformly across its aperture. Now, we use the individually addressable electrodes to apply spatially varying modulation patterns that deflect incident light to different directions. The addressing scheme is demonstrated in Fig. 4A, where distinct voltages can be applied to each of the 32 elements such that the reflectance associated with each group of three antennas can be modulated across the metasurface aperture. To demonstrate this functionality, we apply a series of binary grating patterns with different periods to symmetrically diffract light to three distinct angles. We define *P*_0_ = 3 × 1.4 μm as the period of the addressable electrodes (Fig. 3A), which sets the maximum diffraction angle achievable by our metasurface. Grating patterns with three periods (*P* = 4*P*_0_, *P* = 8*P*_0_, and *P* = 16*P*_0_) are applied to the metasurface with voltages of −60 and 0 V on adjacent groups of electrodes (Fig. 3A).

**Fig. 4.**
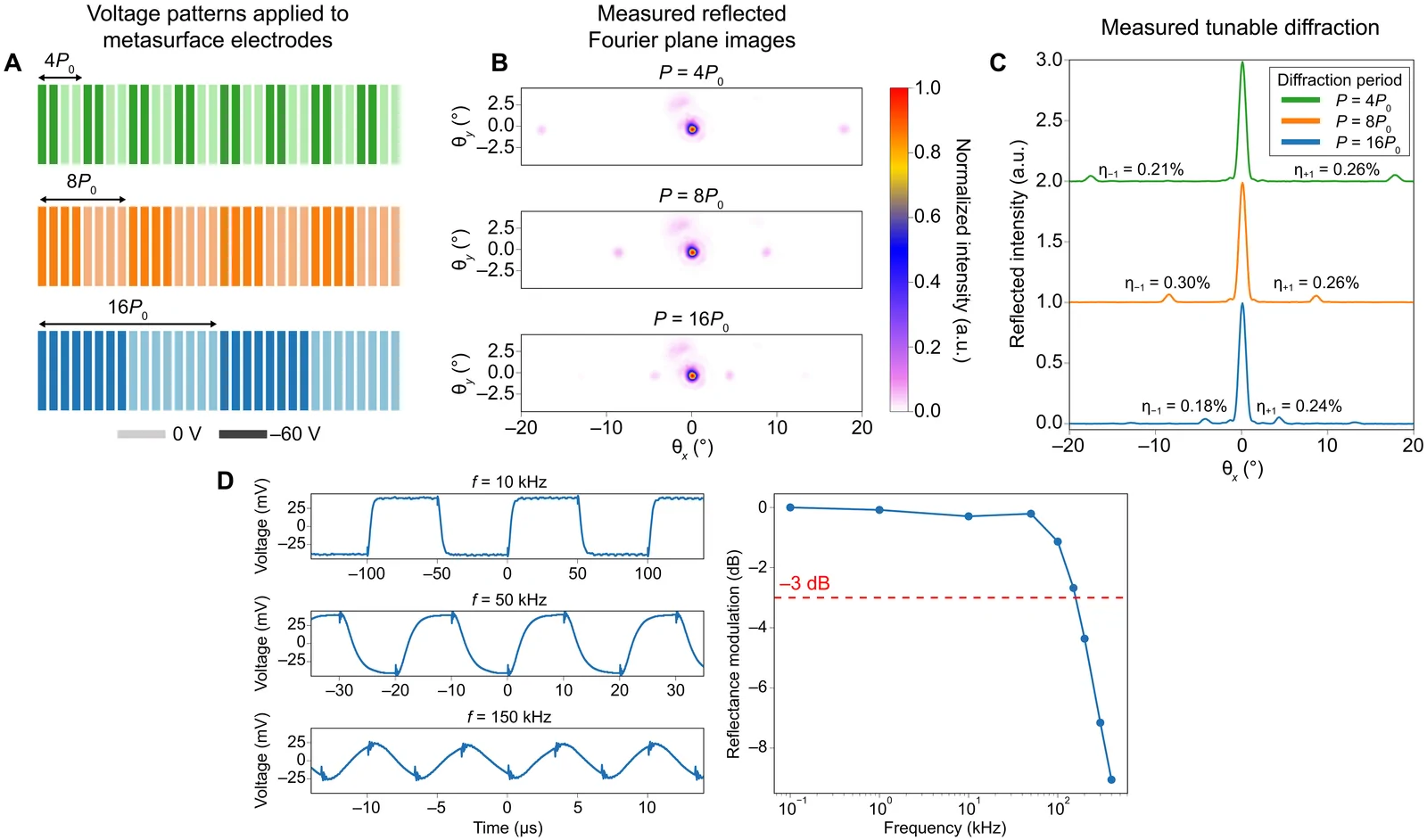
1D diffraction. (**A**) Voltage patterns applied to the metasurface. The lighter and darker shades represent 0 and −60 V, respectively. The period of the metasurface antennas is indicated as *P*_0_. (**B**) Fourier plane images and (**C**) linecuts through θ_*y*_ = 0° for the three voltage patterns in (A). The images are normalized to the zeroth-order maximum in each of the three voltage configurations. (**D**) Measured zeroth-order reflected signal of the metasurface when modulated uniformly at 10, 50, and 150 kHz (left) and reflectance modulation as a function of driving frequency (right).

To characterize the diffraction from the metasurface, we image the Fourier plane using a ZnSe aspheric lens for each grating pattern in Fig. 4A. The experimental setup is detailed in section S4. Figure 4B shows the measured Fourier plane images where we observe clear, symmetric diffraction orders between 4° and 18° for a wavelength of λ = 4.95 μm. We have subtracted a background frame without any illumination, and the images are normalized to the peak zeroth-order intensity. Offset linecuts of these images along θ_*y*_ = 0° are shown in Fig. 4C, and we can see that the intensity of the steered light is ∼5% of the zeroth order. The diffraction efficiencies of the *m* = ±1st orders (labeled in Fig. 4C for each diffraction period) are calculated by referencing the Fourier plane images to the measured reflectance modulation from Fig. 3D. For all orders, the diffraction efficiency ranges from ∼0.2 to 0.3%. While this is certainly low, it is comparable to previous demonstrations of integrated OPAs in the mid-IR (*11*). To perform these measurements, we illuminate the metasurface with ∼100 mW of optical power and thus expect the power in each diffracted order to be between 180 and 300 μW. A detailed comparison between our device and existing methods of beam steering in the mid-IR is presented in section S5. The zeroth-order beam is stronger than the diffracted orders because the resonance modulation is dominated by a change in reflectance. Thus, we expect minimal phase shift between adjacent elements, and, as such, we do not expect to strongly suppress the zeroth order. While simulations indicate a modest amount of phase shift (section S6), in section S7, we present far-field calculations that agree with the measured Fourier plane images and corroborate the lack of phase shift in our devices. Due to small variations between our fabricated and simulated devices (e.g., thickness of each film, carrier concentration of ITO, optical properties of dielectric, and thickness of gold antennas), it is expected that our experimentally realized phase modulation will be less than simulated. Furthermore, by fitting Gaussians to the diffracted orders, we extract their full width at half maximum (FWHM) divergence and find that, for the cases of *P* = 4*P*_0_, *P* = 8*P*_0_, and *P* = 16*P*_0_, the average FWHMs are 1.006°, 0.891°, and 0.790° (see section S8). We compare these values to the theoretical FWHM of an Airy disk for a beam clipped to a 400 μm-diameter aperture and obtain Δθ = 1.029λ/*D* = 0.729°, which is in excellent agreement with the measured divergence of the diffraction orders. The primary challenge in achieving strong phase modulation in our devices is the temperature sensitivity of the ITO film to its environment due to its exceedingly low carrier concentration (and thus higher oxygen content). We find that, for these films, the carrier concentration is more greatly affected by elevated temperatures, especially when in contact with an oxygen-deficient dielectric. This is mitigated in our devices by ensuring that our dielectrics have as few defects as possible by using the high-quality deposition method, ALD, and extensive postdeposition annealing of each dielectric layer. In a device with strong phase shift, the zeroth order can be suppressed, and light can be steered asymmetrically. However, the overall efficiency of the steered light may be comparable to the case of pure amplitude modulation as phase modulation in plasmonic metasurfaces occurs near critical coupling, necessarily introducing stronger absorption (*34*).

We next study the temporal response of the metasurface by driving all electrodes with square wave signals between 1 and 400 kHz with an arbitrary waveform generator. We measure the reflected zeroth-order signal from the metasurface on a HgCdTe photodetector (InfraRed Associates Inc., MCT-5-N-0.25) coupled to a preamplifier (Infrared Systems Development, MCT-1000). In Fig. 4D, the left panel shows the measured reflected signal when driving the metasurface at 10, 50, and 150 kHz. In the right panel, the relative modulation depth is plotted as a function of the driving frequency, and we find that the 3-dB bandwidth is ∼155 kHz. Although ITO-based metasurfaces have been demonstrated to be capable of modulation frequencies in the MHz range, the preamplifier used in our experiments only has a bandwidth of 150 kHz, which ultimately limits the measurable frequency range. However, this suggests that the metasurface itself is not limiting the modulation bandwidth and that it can likely be driven at higher frequencies than what we report here. In section S9, we have included a circuit modeling analysis of the device-limited modulation speed of our driving architecture. We account for the exact geometry of the metasurface elements, the layout of electrical traces, and the bond wires used to interface the device with the driving electronics. We estimate that our architecture may support speeds of up to 32 MHz and propose further improvements that could enable up to 850 MHz modulation.

### Polarization-independent 2D control of diffraction and thermal emission

A fundamental challenge with 2D modulation of active metasurfaces is the increased complexity of the driving electronics and electrical layout to address individual pixels (*38*, *39*). Recently, it has been proposed that a perimeter-addressed array of crossed electrodes (similar to passive matrix driving schemes in displays) can be used to achieve 2D beam steering in an active metasurface given scattering elements with a phase response (*40*). Inspired by this work, we introduce a simple modification to our metasurface architecture such that the ITO layer is patterned into strips, creating addressable electrodes running orthogonal to the gold electrodes below. However, because the response of our devices is amplitude dominated, the kinds of patterns that they can generate are more limited than what is presented in (*40*), where phase-only modulation is required to steer light into a single order at arbitrary polar and azimuthal angles. Specifically, this limits us to diffraction experiments along orthogonal grating directions relative to our electrodes. In contrast, an amplitude-only device with pixel-level addressing could deflect light to other azimuthal angles. For more complex wavefront-modulation applications such as holography, perimeter addressing also imposes additional constraints compared with full pixel-level addressing. A top view cartoon of this configuration is shown in Fig. 5A. The resonant antennas are now circular (producing a polarization-independent resonance) and sit in 12 by 12 arrays atop the regions where the ITO and gold strips overlap. The antennas still maintain a period of 1.4 μm, but, between each group of 12 by 12, there is a 400 nm gap introduced to better isolate the electrodes electrically. Thus, the period of the gold and ITO electrodes is 17.2 μm. These patches of antennas are modulated by applying voltages to the corresponding row and column electrodes. Now that there are additional addressable electrodes along the *y*-direction, the 32 programmable channels are split into two groups of 16, routed to separately control the gold and ITO electrodes that span the entire 275 μm–by–275 μm device aperture.

**Fig. 5.**
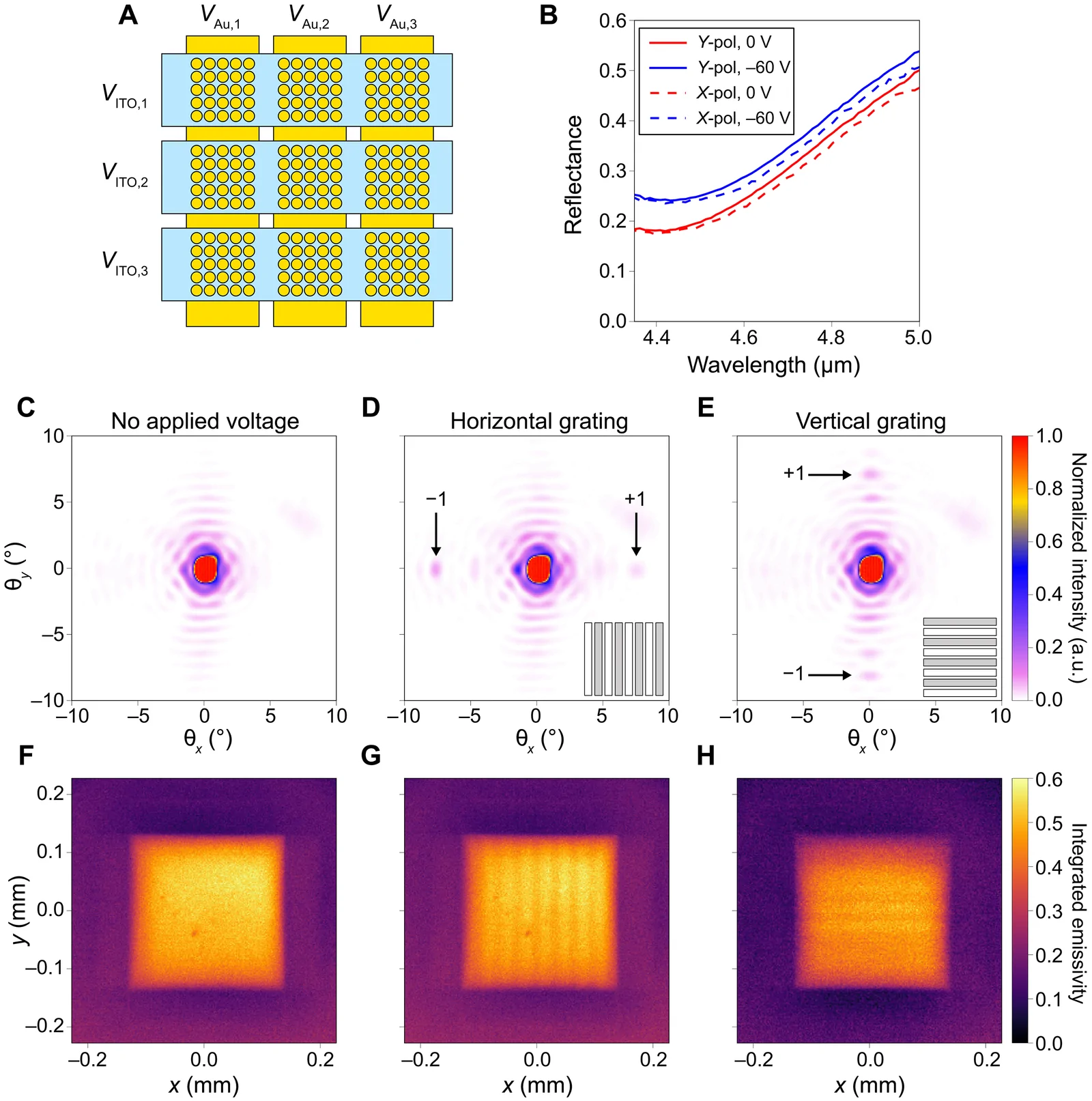
2D diffraction and emissivity modulation. (**A**) Top view cartoon of the crossed ITO and Au electrodes enabling perimeter addressed modulation by applying voltages $V_{\text{ITO},i}$ and $V_{\text{Au},i}$. At the electrodes’ intersections there are clusters of 12 by 12 circular Au antennas (shown here as fewer for simplicity). (**B**) Measured reflectance spectrum for *x*- and *y*-polarized illumination with 0 and −60 V applied between the ITO and Au. The voltage pattern is uniform across the metasurface aperture. Measured Fourier plane images with *y*-polarized illumination at λ = 4.4 μm for (**C**) 0 V on all electrodes, (**D**) alternating 0 V/−60 V on Au electrodes, and 0 V for all ITO electrodes, producing a grating running horizontally, and (**E**) 0 V/−60 V on alternating ITO electrodes and −60 V for all Au electrodes, producing a grating running vertically. The inset diagrams in (D) and (E) show the orientation of the grating patterns. (**F** to **H**) Images of the emissivity across the metasurface aperture captured in the absence of laser illumination for the same three voltage patterns as (C) to (E). The only source of heat is from the PCB upon which the metasurface is mounted, measured to be ∼50°C. The absolute emissivity is obtained by normalizing to a soot sample mounted identically to the metasurface.

First, we study the resonance modulation of this device by measuring its reflectance spectrum with a uniform voltage applied across the aperture. In Fig. 5B, the experimentally measured reflectance spectrum is shown at normal incidence for orthogonal incident polarizations with 0 and −60 V applied across the top Al_2_O_3_ to deplete the ITO. We find that the resonance is centered near λ = 4.4 μm and observe strong modulation of the reflection spectrum for both polarizations. Small differences in the reflectance for orthogonal polarizations are attributed to the patterned ITO and gold electrodes slightly breaking the symmetry that would be present in the case of a uniform antenna array. Next, we probe the ability of our device to steer light in orthogonal directions. With an identical Fourier plane imaging setup as in the previous section, we capture images at λ = 4.4 μm with no voltage applied, a grating pattern applied to the gold electrodes, and a grating pattern applied to the ITO electrodes. The resulting images for *y*-polarized light are shown in Fig. 5 (C to E) and confirm that our device is capable of steering in orthogonal directions, albeit with low efficiency. The zeroth order is saturated in the camera to more clearly view the first-order diffraction spots in all three voltage configurations. The corresponding images for *x*-polarized light are included in section S10. Based on the reflectance spectrum of Fig. 5B exhibiting less relative amplitude change compared with the 1D device, it is expected that the diffraction efficiency should be lower. Another reason for low diffraction efficiency is that some adjacent electrodes were found to be electrically shorted due to fabrication imperfections and needed to be disconnected. As a result, the grating pattern is not perfectly periodic which has a negative effect on the diffraction efficiency and spot size. However, as will be discussed later, the higher overall reflectance translates to a greater relative modulation of emissivity.

The fact that our metasurface operates in the mid-IR affords us the opportunity to explore its potential to spatially modulate thermally emitted radiation. Kirchoff’s law of thermal radiation states that, in equilibrium, an object’s emissivity is equal to its absorptivity, and there have been recent demonstrations of metasurfaces that modulate thermal emission by tuning their resonances (*21*). However, to the best of our knowledge, there has been no demonstration of a spatial light modulator capable of active spatial patterning of thermal emissivity. To investigate this in our metasurface, we image the metasurface (setup shown in section S4) and apply the same three grating patterns as in Fig. 5 (C to E) in the absence of laser illumination. The metasurface reaches a temperature of ∼50°C due to the heat of the driving electronics beneath it. With no additional heat source, we clearly observe the metasurface aperture emitting radiation with an intensity greater than that of the surrounding materials when no voltage pattern is applied (Fig. 5F). Applying horizontally and vertically oriented grating patterns to the ITO and gold electrodes produces visible sets of orthogonal lines in each direction (Fig. 5, G and H). We note that the image in Fig. 5H was taken from a different metasurface than the images in Fig. 5 (F and G) due to inadvertent electrical damage to the latter. The absolute emissivity is obtained by referencing the measured camera counts to those of a soot sample, which we assume approximates a perfect blackbody emitter. The observed lines across our metasurface are a direct result of an emissivity change due to the resonance modulation changing the absorptivity of the metasurface. Missing lines are the result of shorted electrodes being disconnected, as mentioned above. Thermal emission is spectrally broadband so we must consider the spectral origin of the thermal signatures captured. Our camera is only sensitive to wavelengths from 1 to 5 μm, and the Planck blackbody spectrum of a 50°C distribution is centered near λ = 11 μm such that any absorptive resonance at shorter wavelengths contributes minimally to the emissive signal. In section S11, we present the simulated reflectance spectrum spanning our camera’s sensitivity window for two different ITO carrier concentrations and confirm that the primary resonance responsible for emission is the gap plasmon considered in our devices. Thus, we conclude that the source of the patterned thermal emission is a change in emissivity due to modulation of the optical resonance demonstrated in Fig. 5B.

## DISCUSSION

In this work, we have introduced an active metasurface platform for controlling coherent and incoherent mid-IR radiation in one and two spatial dimensions. We rely on postfabrication thermal processing to tune the ITO carrier concentration and precisely control its complex dielectric function to achieve gap plasmon resonances between wavelengths of 4 and 5 μm. In a 1D grating device, we have demonstrated symmetric beam steering with addressable elements of subwavelength spacing based on amplitude modulation across the metasurface aperture with diffraction efficiencies comparable to existing approaches. We then extended this platform to show the diffraction of light in two orthogonal directions with a perimeter addressing scheme that scales well with aperture size. Last, by heating our device, we examined the ability to spatially pattern thermal emissivity across the metasurface aperture at constant temperature.

Throughout this work, the highest diffraction efficiency of our device is limited to a maximum of ∼0.3% due to our use of amplitude modulation. While this efficiency is low, it should be noted that it is comparable to what may be achieved in an ITO-based plasmonic device with large phase modulation as this device would inherently have lower reflectance. Compared with previous demonstrations in the near-IR (*34*–*36*), our 1D steering exhibits larger reflected power in the zeroth order but similar reflected power in the diffracted orders of interest. Furthermore, higher reflectance implies less absorption and thus heating, meaning it is better suited to high-power illumination. We find that the performance of our devices is unchanged with 100 mW of incident optical power, indicating good power handling and minimal induced heating. The total efficiency could be improved by using techniques previously studied in the near-IR such as using two applied biases per unit cell to avoid operation near critical coupling (*34*) or by working with other materials such as CdO, which exhibit lower intrinsic optical loss (*41*). Overall, while we have addressed several requirements for a mid-IR spatial light modulator, there is still room for further optimization specifically in terms of diffraction efficiency and phase modulation, which we highlight as important future research directions.

In evaluating an active optical element, it is also important to consider electrical energy consumption and reliability performance. In our 1D device, we measure ∼10 nA leakage current when applying the maximum possible voltage of 60 V to all electrodes. Given our device area of ∼400 μm by 400 μm, this corresponds to a leakage current density of ∼6.25 μA/cm^2^. The switching energy for each addressable unit cell is also an important metric. To approximate this, we model an addressable unit cell of our 2D device as a parallel plate capacitor (*E* = ^1^/_2_ CV^2^) with area 17.2 μm by 17.2 μm. Using a dielectric permittivity for Al_2_O_3_ of 9 (*42*) and a dielectric thickness of 160 nm, we obtain a switching energy of 265 pJ per unit cell and an energy density of 5.60 × 10^−21^ J/nm^3^, which is lower than previously reported switching energies for low-energy switching devices based on phase change materials (*43*). Regarding device reliability, during the acquisition of the switching speed measurements presented in Fig. 4D, our device underwent over one million electrical cycles with no degradation of modulation. We do highlight, however, that our device can be prone to electrical shorting due to our maximum voltage operation achieving electrical fields of ∼3.75 MV/cm, which approaches the reported breakdown field of ∼8 MV/cm for similarly grown Al_2_O_3_ films (*44*). Assuming that our device architecture is perfectly planar across the whole device aperture, we would be sufficiently far from the breakdown field. However, due to the complex device architecture requiring our gate dielectric to conformally coat many gold electrodes, it is to be expected that some areas of the device will have thinner dielectric and cause applied electric fields to be even closer to the breakdown field. Such reliability concerns, however, could be mitigated in commercial fabrication settings by reducing unwanted contamination and using planarization steps to reduce roughness of each layer.

Looking forward, we envision the potential for fine-tuning our metasurface structure to achieve stronger phase modulation. This would enable highly directive and asymmetric steering of an incident beam in two spatial dimensions via perimeter addressing, which is not possible with pure amplitude modulation. Additionally, our work may ultimately lend itself to coherent control of thermal emission, whereby a nonlocal mode is engineered to extend the spatial coherence of the emitted thermal radiation and couple adjacent scattering elements. We suspect that our results may open previously unexplored opportunities for active devices in the mid-IR, a spectral region often overlooked yet replete with exciting applications.

## MATERIALS AND METHODS

### Device fabrication

Fabrication began on a 2-inch (5.08-cm) <100> silicon wafer with a 1 μm thermally grown oxide. The back gold layer was patterned using electron-beam lithography (EBL), deposition using electron-beam (e-beam) evaporation of 5 nm Ti (for improved adhesion) and 80 nm Au, and then liftoff in Remover PG. The EBL system used was the Raith electron beam pattern generator (EBPG) 5000+ and the e-beam evaporator was the Kurt J. Lesker Labline Electron Beam Evaporator. Next, we patterned 200 nm of Al_2_O_3_ via ALD using a shadow mask. The ALD system used was the Ultratech/Cambridge NanoTech Plasma Enhanced ALD System Fiji G2. After the Al_2_O_3_ deposition, the whole wafer was annealed on a hotplate at 200°C in air. Next, another gold layer was patterned using EBL, e-beam evaporation of 5 nm Ti and 80 nm Au, and then liftoff in Remover PG. This layer formed the bottom electrical interconnects for both 1D and 2D devices. After this, another masked ALD deposition of 160 nm Al_2_O_3_ was deposited via ALD. Next, 50 nm of ITO was patterned using EBL, sputtering using an AJA International RF magnetron sputterer with a deposition pressure of 3 mTorr and RF power of 100 W while flowing 20 standard cubic centimeter per minute (sccm) pure Ar and 20 sccm Ar/O_2_ in a 90/10 wt % mixture and then liftoff in Remover PG. For the 1D case, the ITO was patterned into a continuous film across the active metasurface area. For the 2D case, the ITO was patterned into individual strips to allow for separate biasing. At this point, we then performed multiple anneals of our sample at 150°C in air and vacuum until we measured the desired carrier concentration of our ITO film. Annealing in air decreased carrier concentration, and annealing in vacuum increased the carrier concentration. We characterized the carrier concentration through ellipsometric measurements and Drude-Lorentz model fits with a wavelength range from 1.5 to 20 μm. Once the desired ITO carrier concentration was obtained, we lastly patterned Au antennas using EBL, e-beam evaporation, and liftoff in Remover PG. For this final step, all baking steps were performed at 100°C to prevent altering the ITO properties. Additionally, we did not use a Ti adhesion layer to avoid any reaction between the Ti and ITO film.

### Measurements

All optical measurements were performed using a homebuilt setup. Section S4 describes this setup in more detail.

### Device simulations

All optical simulations were performed using Ansys Lumerical FDTD Solutions. Charge carrier simulations were performed using the Ansys Lumerical CHARGE module (see section S2).

## Supplementary Materials

### The PDF file includes:

Sections S1 to S11 Figs. S1 to S20 Tables S1 and S2 Legends for movies S1 and S2 References
