# Supplementary material for: Electro-optic modulation of coherent and incoherent mid-IR radiation in two-dimensional arrays
Source: Sci Adv. 2026 Sep 25;12(39):eaeg3189. doi: 10.1126/sciadv.aeg3189 (PMC13614366; doi:10.1126/sciadv.aeg3189)
Supplement: Supplementary file 1 [file sciadv.aeg3189_sm.pdf]

Science Advances
AAAS

Supplementary Materials for

Electro-optic modulation of coherent and incoherent mid-IR radiation in two-dimensional arrays

Jared Sisler *et al.*

Corresponding author: Harry A. Atwater, haa@caltech.edu

Sci. Adv. **12**, eaeg3189 (2026)
DOI: 10.1126/sciadv.aeg3189

The PDF file includes:

Sections S1 to S11
Figs. S1 to S20
Tables S1 and S2
Legends for movies S1 and S2
References

Other Supplementary Material for this manuscript includes the following:

Movies S1 and S2

Supplementary Text

Section S1 – Evaluation of crosstalk in device

Due to the strong optical field localization of gap plasmon resonances, our device exhibits very little crosstalk between adjacent active electrode groups. Figure S1a plots the simulated optical field for a group of three antennas, showing the strong localization of electric field underneath the gold antennas. Additionally, Fig. S1b shows the simulated electrostatic field for two antenna groups (three antennas per group) when the maximum and minimum voltage is applied (0 V and –51 V). In this plot, the electrostatic field is very uniform directly underneath each antenna. While there are some fringing fields in between the gold electrodes, this does not affect our final modulation because of the strong optical confinement beneath the gold antennas (Fig. S1a). In addition, the excellent agreement between the measured diffraction profiles and those predicted from array factor calculations, which assumes zero coupling between scattering elements, (Section S7) provides further evidence that our device exhibits very little crosstalk between active elements. This is a strength of our device as crosstalk is a fundamental drawback for many active optical elements such as liquid crystal-based spatial light modulators and has limited the miniaturization of addressable pixel sizes.

Section S2 – Device charge transport simulations

We present the simulated electron charge density profile throughout our ITO films under an applied bias. Our simulations were completed in Ansys Lumerical CHARGE module and consist of a 50 nm film of ITO on top of a 200 nm film of Al_2O_3, sandwiched between to electrical contacts. The electrical permittivity of Al_2O_3 is assumed to be 9. The ITO electron mobility is assumed to be 30 $cm^2/(Vs)$, the effective mass 0.3, and the carrier concentration was changed between $3x10^{20}$ cm^{-3} and $3x10^{19}$ cm^{-3} to provide a comparison. These two values were selected as they represent a typical carrier concentration used in ITO-based active plasmonic devices in the near-IR ($3x10^{20}$ cm^{-3}) (*33,35*) and the carrier concentration used in this work ($3x10^{19}$ cm^{-3}). The voltage applied between the two electrical contacts was 50V (Fig. S2a) and -50 V (Fig. S2b) relative to the ITO/contact interface such that there was carrier accumulation/depletion in the ITO at the Al_2O_3/ITO interface. Figure S2a plots a 1D cross-section of the carrier concentration as a function of distance from the Al_2O_3/ITO interface for the two different bulk carrier concentrations of ITO under an applied bias of 50 V. The accumulation region widths are similar between the two carrier concentrations, with the $3x10^{19}$ cm^{-3} case being slightly larger its charge density is lower. For both bulk carrier concentrations, the accumulation region width is less than 5 nm. Figure S2b shows the charge profile when a voltage of -50 V is applied and the ITO is forced into depletion. Here, we can see the benefit of using charge depletion instead of accumulation for the lower carrier concentrations used in this work. Since the bulk charge is so much lower, the depletion region must be much larger in order to compensate for the voltage applied to the device. The depletion region associated with the $3x10^{19}$ cm^{-3} case is approximately 10 nm and is much larger than its associated accumulation width. Thus, by operating in depletion, we can make use of the electrical and optical modulation in a larger volume of ITO. For previous works in the near-IR the different between the accumulation and depletion widths are not as significant due to the larger bulk carrier density.

It should be noted that the simulations performed here assume the classical drift-diffusion model and simply look for general trends between different bulk carrier concentrations. It has been reported that devices with confined dimensions (on the order of the electron wavelength) can be

more accurately modeled using the Schrödinger-Poisson (SP) quantum model to calculate the carrier density (*45*). The main difference between the two models is that the classical drift-diffusion model allows for unphysically high concentrations of carriers, as can be seen in Fig. S2b where the electron concentration extends sharply towards zero. This would result in an extremely high concentration of holes at the ITO/Al_2O_3 interface in our device which is not allowed due to the quantum filling of the available states. Figure S2b is intentionally cut-off as the simulated carrier concentration values become unphysical near the ITO/Al_2O_3 interface. Despite this inaccuracy, we can still use our classical simulations to provide an estimate of the depletion width to be used in our full-wave optical simulations of reflectance modulation.

Section S3 – Additional images of devices

We present additional images of our devices taken by optical microscope, optical camera, and scanning electron microscope. Figure S3 shows optical microscope images of our 1D and 2D devices which show the active area in the center and the 32 electrical interconnects spreading out form the center. Figure S4 shows SEM images our 1D active metasurface device and Fig. S5 shows our 2D active metasurface device. In each case, one can see in more detail how the electrical interconnects address the grouped arrays of antennas which are precisely aligned on top of each contact. In both 1D and 2D devices, the spacing between the electrical contacts is very small, approximately 50 nm. This is most easily seen in Fig. S4f and Fig. S5e. This small electrical isolation allows us to not significantly perturb the periodicity of the antennas but makes our device sensitive to electrical shorting.

Section S4 – Experimental optical setup for metasurface characterization

The experimental setup used to perform the measurements presented in this work is shown in Fig. S6. It is used in three configurations (1) for measuring reflectance spectra, (2) capturing Fourier plane images, and (3) imaging the metasurface plane for spatially-resolved emissivity measurements. A free-space tunable quantum-cascade laser (QCL, Daylight Solutions) is used to illuminate the metasurface for configurations (1) and (2). A lens L1 weakly focuses the beam to the back focal plane (BFP) of L2, which roughly re-collimates the beam as it illuminates the metasurface. The beam diameter is controlled by an adjustable iris. In configuration (1), L3 is removed from the beam path such that the light reflected from the metasurface is directly collected by a thermopile detector and the camera is conjugate with the metasurface plane. In configuration (2), L3 is in place which makes the camera conjugate with the BFP of L2. Then, to capture emissivity measurements are performed with the laser off and L3 removed from the optical path. This way, we directly capture the thermal emission in the metasurface plane.

Section S5 – Comparison to existing mid-IR beam steering devices

Here we present a comparison of our work against other steering platforms in the academic literature and those that are commercially available. This analysis is summarized in Table S1 along with relevant references. First, we consider beam steering solutions with mechanical moving parts such as galvo mirrors, scanning micro-electro-mechanical system (MEMS) mirrors, and Risley prisms (*46*, *47*). These platforms are highly efficient because they are limited by either the reflectance of the mirror or the transmittance through a pair of prisms, which can be coated for high efficiency. A primary drawback of these platforms is slow scanning speed due to bulky elements such as prisms and mirrors. Furthermore, scanning mirrors often operate resonantly to reduce their power consumption, which limits the scanning patterns to be of Lissajous-type and

precludes fast, random-access steering. Another example of a mechanical beam-steering platform in the mid-IR is a digital micromirror device (DMD). These devices consist of an array of MEMS tip-tilt mirrors that can be switched such that they provide spatially patterned amplitude modulation. As mentioned in the main text, the pixel pitch of such devices typically ranges from few to tens of microns (*9*, *10*). As pixels tend to the larger end of this range, the steering angles and diffraction efficiencies become limited. Evidently, there are several mid-IR beam-steering solutions based upon mechanically moving parts. However, they all suffer from relatively slow steering speeds and are susceptible to mechanical failure.

Non-mechanical beam-steering in the mid-IR is comparatively sparsely populated. For visible and near-IR wavelengths, liquid crystal (LC) based phase only spatial light modulators (SLMs) are ubiquitous and provide high efficiency steering with high spatial resolution. However, at mid-IR wavelengths between 4–5 μm, many LCs are strongly absorbing and, as a result, to the best of our knowledge there are currently no commercially available LC-based SLMs in this spectral range. There are two examples in the literature of steering devices based on LCs. In one instance, a device compatible with diffractive switching in the mid-IR was introduced, but switching was not experimentally demonstrated, although the authors were able to achieve a diffraction efficiency of 98% for a static LC grating (*5*). The other instance consists of a slab waveguide with a LC overcladding with electrodes configured such that light is deflected laterally within the slab (*48*). Here, the diffraction efficiency is 3%, but requires up to 500 V to drive. Another class of beam steering-devices (that are also commercially available) is acousto-optic deflectors (AODs), which deflect light by driving an acousto-optic crystal with RF signals to steer light into one or multiple beams. The diffraction efficiencies of AODs are typically above 80% and their switching speed is on the order of microseconds, but they typically require tens of watts of RF power to operate and are bulky. Finally, integrated optical phased arrays (OPAs) consist of waveguide arrays with individual phase modulators that are terminated with grating couplers to scatter the phase-modulated light to free space. The highest reported diffraction efficiency for an OPA in the mid-IR is 1.5% and a 2D steering device was shown to have a diffraction efficiency of 0.3% (*11*, *49*). These values are comparable to what we obtain in our 1D steering devices. Aside from OPAs, there has also been a steering demonstration in the mid-IR where a near-IR beam, steered from an AOD operating at this wavelength (λ = 1 μm), was downconverted in a nonlinear crystal to λ = 3.5 μm, reaching a total conversion efficiency of 2.8% (*50*). This way, the near-IR AOD could effectively steer the converted mid-IR light.

As the above discussion has shown, there is a dearth of non-mechanical beam steering platforms in the mid-IR. Although the diffraction efficiency of our 1D steering device is low relative to mirror-based devices or commercially available AODs, it is comparable to other experimentally demonstrated non-mechanical platforms such as OPAs. However, diffraction efficiency is not the only metric of interest in steering devices. The power consumption, modulation speed, and number of resolvable spots (N_{RS}) are critical metrics to evaluate the suitability for a given application. N_{RS} is defined as the number of distinct steering angles within the full steering range such that adjacent beams do not overlap. In other words, it is the full steering range of the first order divided by the beam divergence. For commercially available mid-IR AODs N_{RS} can be up to 400 but in the academic literature there has been no demonstration of a platform capable of exceeding 50 resolvable spots. This is owing to the lower pixel density of existing mid-IR OPAs where the minimum separation to avoid thermal and optical crosstalk is chosen to be 2.5λ (or 9-10 μm), compared to 0.85λ for our 1D device. In our device, the full first-order steering range is 2 x 36° (given by the electrode width of 4.2 μm and wavelength of λ = 4.95 μm) and the

average divergence of the diffraction orders is 0.895° which gives $N_{RS} = 80$, the highest reported in the literature for this wavelength range. However, we cannot utilize the full N_{RS} because of the tiled architecture (i.e. repeating the 32 addressable electrodes three times) we utilize to reduce the complexity of the driving electronics. This choice limits our device to approximately $N_{RS} = 32$; the tiled aperture provides the advantage of a reduced beam divergence by way of forming a larger aperture. To highlight this, Fig. S7 shows the grid of adjacent resolvable spots from our device. With more sophisticated driving electronics with addressable channels spanning the entire device aperture, the number of resolvable spots may be dramatically increased and made to be competitive with existing AOD solutions, but we leave this for future research.

Another benefit of our platform is its fast switching time and low power consumption for static operation. Given 60 V applied to all electrodes of our device, the leakage current is below 10 nA and thus the static power consumption to drive the entire array is below 600 nW. In comparison, experimentally demonstrated OPAs with integrated heaters require at least 100 mW of electrical power to shift the phase by π radians such that driving the entire OPA for arbitrary steering angles may consume up to 1W of power, many orders of magnitude more than our devices. Similarly, commercial AODs can require between 1 W to 100s of W to operate at their peak diffraction efficiencies. Regarding switching speed, the OPAs are limited to ms response times due to the thermal settling time of the phase shifters. Conversely, AODs are much faster with typical switching times between 1-10 µs depending on the beam diameter. We have demonstrated in the main text that our devices can switch at speeds near 150 kHz (limited by the bandwidth of the detector and amplifier), corresponding to a time scale near 6.6 µs, on par with AODs.

In conclusion, our analysis leads us to believe that while the efficiency of our device is low compared to bulky mirror or prism-based solutions, when compared to non-mechanical beam steering platforms in the mid-IR our platform presents several compelling benefits such as many resolvable spots (with a clear path for scaling), minimal power consumption, and fast steering. The table presented below summarizes the above discussion and situates our device among other mid-IR beam steering platforms in the academic literature and available commercially, to the best of our knowledge.

Section S6 – Simulated phase shift of active metasurface

Figure S8 shows the simulated phase shift of our device in simulation. Each curve plots the simulated spectral phase response for an assumed carrier concentration in an assumed 10 nm depletion region of ITO at the ITO/Al_2O_3 interface. The maximum phase shift (difference between the $3x10^{19}$ cm^{-3} and $1x10^{19}$ cm^{-3} curves) is approximately 40° and occurs at $\lambda = 4.85$ µm. We emphasize here that this phase shift was not critical to our device design and in experiment we expect all modulation to come from amplitude modulation.

Section S7 – Array factor calculations of far-field diffraction profile

In order to evaluate the experimental performance of our tunable diffraction and to gain insight into any possible phase modulation which may exist within our device, we present a detailed investigation of the expected far-field spatial diffraction profiles in experiment and simulation.

We start our analysis by extracting the maximum measured amplitude contrast for our 1D metasurface. This corresponds to the 0 V and –51 V curves in Fig. 3d of the main text. We then construct binary 1D gratings with these amplitudes (for each wavelength) in a 288x288 array (corresponding to a ~ 400 µm x 400 µm array assuming one cell is 1.4 µm x 1.4 µm), then apply a

400 μm diameter circular aperture to each array of field amplitudes. This recreates the reflection amplitudes that exist in the measurement of our 1D metasurface. Then, by taking a 1D linecut through the center of the magnitude squared of the 2D Fourier transform of this amplitude array, we obtain the expected far-field intensity profile from our metasurface. This is plotted (Fig. S9a) for the 3 different periodicities and wavelength used in the measured diffraction profiles presented Fig. 4c of the main text. The two plots show good agreement when comparing peak width and height. In measurement, the intensity of the P = 8P_0 diffracted orders is approximately 4.2% the intensity of the 0th order and have a Gaussian-fitted FWHM width of ~0.90°. In our calculated diffracted profiles for the P = 8P_0 periodicity, the intensity of the diffracted orders is approximately 3.6% the intensity of the 0th order with a Gaussian-fitted FWHM width of ~0.73°. This excellent agreement indicates that the measured diffraction in our metasurface is purely a result of amplitude modulation and there is no additional phase modulation exhibited by our device. It should be noted that the fitted FWHM for our array factor calculations agrees with the theoretical FWHM from a circular aperture: $\theta_{FWHM} = 1.029\lambda/D = 1.029 \times 4.95\ \mu\text{m}/400\ \mu\text{m} = 0.73°$. Thus, it is expected that our measured width will be slightly larger than this due to our aperture being slightly smaller than 400 μm. In experiment, we manually close an iris until the edges of our metasurface are covered. Thus, the experimental aperture will be slightly smaller than 400 μm, causing peak broadening. Additionally, any aberrations in our imaging system will also increase the FWHM of our measured peaks.

Next, we repeat this array factor calculation for each wavelength in Fig. 3d of the main text and extract values from the far-field profiles. Figure S9b plots the measured relative amplitude modulation, as a function of wavelength, for our 1D device. Here, we see that the largest relative modulation occurs at the measured wavelength in Fig. 4c of the main text, λ = 4.95 μm. Similarly, Fig. S9c takes the ratio of the calculated peak intensity in the 1st order to the peak intensity in the 0th order. This curve closely follows the trend of relative amplitude modulation in Fig. S9b. Finally, Fig. S9d estimates the total diffraction efficiency by integrating the intensity in both diffraction orders, dividing by the total integrated intensity, and multiplying by the average reflectance across the aperture. The trend shown in Fig. S9d differs from the trend in Figs. S9b,c because even as the relative amplitude modulation decreases, the overall reflectance increases. While there is a tradeoff between these two metrics, the maximum diffraction efficiency is still maximized at approximately the same wavelength.

As a comparison, we provide the same array factor calculations, this time using the simulated optical modulation of our device. The amplitude modulation is taken from Fig. 3c in the main text and the phase modulation from Fig. S8 in the Supplementary Materials. Using these simulated values, we see the far-field diffraction patterns (Fig. S10a) look similar to the experimentally predicted (Fig. S9a) and measured (Fig. 4c in the main text) patterns. Thus, we can conclude that our experimentally realized device does not vary significantly from our design. The maximum amplitude and phase modulation used for these array factor calculations are summarized in Fig. S10b showing similar relative amplitude modulation to our experimentally measured curve. Figure S10c plots the ratio of intensities in the 1st to the 0th orders, showing a similar trend to the experimentally predicted curve, but overall higher ratios. The higher intensity of the 1st order relative to the 0th order is caused by the small amount of phase shift in the simulated calculations. Finally, the overall diffraction efficiency in both diffracted orders is plotted in Fig. S10d. Again, the trend and absolute values in this plot are very close to those measured and predicted through array factor calculations using experimentally measured modulation.

The analysis in this section raises an interesting point regarding the total diffraction efficiency of active metasurface devices. Namely, the relative intensity of diffraction orders to non-diffracted orders can be approximated by relative amplitude and/or phase modulation curves. However, to gain insight into the final diffraction efficiency, the absolute reflectance (or transmittance) values must be considered important. This is an important consideration for phase modulation devices which rely on passing through a point of critical coupling. While the capability for large phase modulation enables many functionalities such as asymmetric diffraction, it does not necessarily result in higher diffraction efficiencies. Additionally, for a reflective device operating near critical coupling, absorption is necessarily very high, which can result in unwanted heating and device degradation.

Section S8 – Divergence of diffraction orders

We quantify the angular divergence of the diffraction orders by fitting Gaussians to the central region of the diffraction peaks and extracting their FWHM values for all three diffraction angles in Fig. 4 of the main text. Fundamentally, the angular divergence of limited by the size and shape of the device aperture. In our case, we relay an adjustable circular iris that is uniformly illuminated onto the 400 μm x 400 μm metasurface aperture with a wavelength of λ = 4.95 μm. Thus, we model the beam divergence based on the formula for diffraction from a circular aperture given by $\Delta\theta$=1.029λ/D where D is the aperture diameter. For our device, this corresponds to a theoretical FWHM divergence of 0.729°. The diffraction order fits to experimentally measured Fourier plane images are shown in Fig. S11, which presents the same data as in Fig. 4 of the main text, but with the fit curves and corresponding extracted FWHM divergences labelled. The average FWHMs for diffraction periods of P = $16P_0$, P = $8P_0$, and P = $4P_0$ are 0.790°, 0.891°, and 1.006°, respectively. These values are in good agreement with the theoretical divergence computed above, particularly for the largest period. The measured divergences increase monotonically with diffraction angle, which is to be expected based on aberrations in the imaging system further away from the optical axis such as coma and spherical aberration.

Section S9 – Driving electronics design and speed simulations

Device biasing circuitry

To enable electrical biasing of our experimental devices at moderate speeds, we developed a custom 32-channel biasing printed circuit board (PCB) based on the following key components:

- Two Analog Devices AD8113 cross-point switches, which provide low-impedance drive buffering with ±10 V output span. Slew-limited full-scale analog output bandwidth is ~3 MHz. The input-to-output pin mapping (i.e., MUX) can be reconfigured synchronously at up to ~250 kS/s. Note that output voltage range can be shifted by applying a separate substrate bias (e.g., applying −10 V substrate bias to achieve output span of 0 to 20 V).
- A bank of manual input selection switches, to select between either (8) 50 Ohm coaxial input jacks for passing full-bandwidth signals from external function generators, or (32) slower on-board DAC channels.
- Two Linear Technology LTC2688 digital-to-analog converters (DACs), providing (32) independent bias voltages with 12-bit precision over the above output span (~5 mV resolution). Full-scale analog bandwidth is ~80 kHz, but synchronous arbitrary waveform

programming speeds are limited to ~25 kS/s. However, a special two-state toggling mode allows faster toggling between two specific bias configurations.

- Two Analog Devices AD7616 analog-to-digital converters (ADCs), providing 16-bit voltage readback for each device pin at up to ~100 kS/s (staggered sampling via four physical ADC cores). Note that ADC measurements were not employed in the present study due to use of eternal amplifier boards; pin voltages were occasionally probed with an oscilloscope to confirm intended sequence programming.
- An ARM Cortex-M7 microcontroller running at 600 MHz (Teensy 4.1 Development Board) to provide a programmatic interface for configuring the above peripherals via USB serial commands from a lab PC. The 1024 kB of processor memory allows storage of DAC, ADC, or MUX tables totaling ~4k rows, which can be used to execute DAC/MUX reconfiguration sequences (and/or trigger and store ADC measurements) with precise timing at up to the above-listed rates.
- 36-pin chip carrier boards (32 bias channels + 4 substrate pins) for mounting experimental devices to the socket on the main PCB. We affix the devices to these smaller boards and wire-bond to the electrodes.

A condensed schematic of the circuit topology is shown in Fig. S12. Power is provided by ±12V and +5V rails via benchtop power supplies. Typical power consumption is ~2.5 W idle and ~4 W during DAC bias sequencing. Images of the circuitry with an unpopulated chip carrier are shown in Fig. S13. Approximate configuration rates and analog bandwidths for relevant modes are shown in Table S2.

To enable higher bias voltage ranges needed for some devices in this study, an additional amplifier interposer board was constructed. This is installed between the main PCB and the chip carriers, mating to both original sockets. It comprises (32) LTC6090 operational amplifiers, each configured for a non-inverting gain of 3.0, powered from ±32V rails, thus increasing the full-scale DAC output range to ±30V, with nominal DAC precision of ~15 mV. The amplifiers add ~5W of quiescent power consumption. We did not explicitly calibrate the resulting circuitry, but cursory analysis of tolerance, offsets, and temperature coefficients suggests that overall error should be <1 LSB of DAC precision in operation. We further confirmed full- and mid-scale output accuracy of each device pin with a digital multimeter prior to measurements.

With 21 V/s slew rate, the amplifier interposer board significantly reduces the analog bandwidth for biasing the devices. The maximum nominal full-scale sinusoidal output frequency is ~100 kHz. For arbitrary step waveform output, ~assuming 50% minimum nominal dwell on bias is desired, waveform rate should be limited to ~80 kS/s. A representative large-signal amplifier output waveform is shown in Fig. S14.

Electrical simulations of limiting device speed

The metasurface devices are in principle capable of faster operation than our experimental equipment can apply bias voltages or observe optical response. To estimate the limiting electrical speed of the devices, we have performed simple circuit modelling, taking into account the distributed inductance, capacitance, and resistance of an individual bias channel within the device, as well as parasitics associated with the fanout and bond wire. This estimates how quickly a bias

voltage signal can propagate into and across the device, but does not address more complex issues such as channel-to-channel coupling.

A substantially simplified diagram and cross-section of the modeled experimental device is shown in Fig. S15. We consider a single bias channel only, starting with the bond wire, which we assume is driven with a 50 Ohm voltage source from its far end. (We do not consider the effects of the chip carrier board or socket, but the bond wire and pad are fundamentally required to bias the device as fabricated.) The assumed bond wire is a 1 cm long, 40 µm diameter aluminum cylinder (L = 10 nH). We model the bond pad as a parallel-plate capacitor, with capacitance calculated based on the pad dimensions (1250 x 250 µm), oxide thickness, and wafer thickness and doping (C = 11 pF). The first portion of the fanout trace is modeled as a 20 µm wide microstrip transmission line[1] atop the oxide / Si wafer, with 3 mm typical overall length. The trace then transitions to a 1.0 µm wide stripline[2] geometry as the fanout approaches the aperture, with the conductor now sandwiched between a lower Au ground plane, and an upper ITO ground plane.[3] A typical inner fanout trace length is 600 µm. Then, the trace becomes a 3.7 µm wide stripline as it transits the active metasurface aperture, which is 400 µm in length. In our experimental devices, we then used 'spiral' traces to route and replicate the bias row sequence two more times across the aperture, using 1.0 µm wide traces. (Thus, our 32-channel experimental devices have 96 rows, repeating the same 32-channel bias sequence three times across the aperture width.) The first spiral trace has a typical length of 1750 µm; the second, 1150 µm.

The above elements were modelled in SPICE to analyze the AC frequency response and time-domain step response of the bias signal propagation. The simulation schematic is shown in Fig. S16. Simulation results appear in Fig. S17.

The bond wire and pad produce a -3dB frequency of 260 MHz, and the fanout traces further reduce this to 60 MHz at the input side of the first antenna bias trace. The antenna bias trace does not substantially reduce the signal bandwidth reaching the opposite side of the aperture (see "Fanout" vs. "Row 1" traces in Fig. S17). The signal then enters the relatively long and narrow spiral trace routing it to the second antenna bias trace, at which point the −3dB frequency is reduced to 32 MHz. There is little further reduction in -3dB bandwidth as the signal traverses the remaining antenna bias traces and spiral trace.

It is evident from this analysis that the bias signal bandwidth is predominantly limited by the wire bond, the fanout, and the spiral traces used to replicate/expand the width of the device aperture, rather than the cutoff frequency for voltage propagation across the active elements themselves. To estimate what reasonable speed could be achieved by addressing these limiting factors, we consider the following revisions:

---

[1] For the various transmision lines' parametric models, we utilized Wcalc (Dan McMahill), accessed at https://wcalc.sourceforge.net

[2] For the asymmetric striplines, we obtained L and C values using RF2DFieldSolver (Jan Käberich), accessed at https://github.com/jankae/RF2DFieldSolver

[3] We do not consider the effect of ITO resistivity here. The calculated microstrip/stripline parameters only consider the resistance of the signal trace (80 nm Au). Field fluctuations caused by ground plane current flow within the ITO is of potential relevance to device operation, but this is beyond the scope of present modelling. We note that the ITO plane grounding could be reinforced with metallization around the aperture perimeter, and in any case, that skin effect is irrelevant for either conductor until frequencies approach ~1 THz.

- The coverage of the ground Au plane and ITO plane is restricted to the active aperture area only, reducing capacitive loading of the fanout traces. The inner fanout trace is now modelled as a microstrip transmission line, similar to the outer fanout trace.
- The inner fanout trace is increased from 1.0 μm to 3.7 μm wide, to reduce series resistance (now equal to the antenna bias trace width).
- The spiral traces and element replication are eliminated; instead, each antenna bias trace would be fanned out to a dedicated wire bond pad. Thus, for an identical aperture width, three times more bias channels would be required (or, three bonds could be placed to connect three antenna bias rows to a single bias input).
- The bond pads are reduced from 1250 x 250 μm in size, to 400 x 200 μm to reduce parasitic capacitance and allow tighter packing. This would be more challenging but perhaps not insurmountable to wire-bond with our equipment.
- The assumed source impedance is reduced to 30 Ohm, which matches the impedance of the simulated circuit at its resonance frequency (~1 GHz).

These proposed changes do not change the wire bond length, fanout trace length, or aperture length of the original circuit. The simulation results for the revised design are shown in Fig. S18. As there is only one antenna bias trace, we separate the response into that at the bond pad, that after the outer and inner fanout traces, and that at the far end of the antenna bias trace. Compared to the signal at the end of the first antenna bias trace of the original circuit, 3dB bandwidth is improved to 850 MHz, and the time to settle to within 2% of a voltage step is decreased from 17.6 ns to 1.4 ns. Even in this case, the speed is largely limited by the fanout and wire bond. It is important to note that this discussion relates only to electrical response, not functional speeds of optical devices. This could vary considerably by application, depending on what degree of phase control precision (i.e., how much voltage settling time) is required.

Section S10 – Polarization dependence of diffraction in 2D

We present the measured diffracted light from our 2D active metasurface for x-polarized illumination. Figure S19 presents the Fourier plane images using all the same parameters as is presented in Fig. 5 of the main text, except for the incident illumination polarization being rotated 90°. The diffraction profiles look essentially identical to those in Fig. 5 of the main text for the opposite polarization and verify our device's insensitivity to polarization enabled by the circular antennas.

Section S11 – Analysis of measured radiance modulation

In Fig. 5 of the main text, we image an actively reprogrammable change in the emission of our metasurface across its aperture. Here, we show that this change in emission intensity corresponds to the change in emissivity at the designed resonance wavelength of approximately λ 4.4 μm. In Fig. S20a, we plot the calculated spectral radiance of a blackbody at 50 °C, the measured temperature of our metasurface during measurement, using Planck's Law. The radiance peaks around $\lambda = 16$ μm and drops off sharply for wavelengths shorter than $\lambda = 10$ μm. The camera used in our experiments has a band edge corresponding to a wavelength of $\lambda = 5$ μm and we plot the corresponding responsivity window as a shaded red box in Fig. S20a. Thus, due to the band edge of our detector, we can be sure that we are not detecting any change in emission from our device that may be occurring at wavelengths longer than $\lambda = 5$ μm. Additionally, as the radiance at 50 °C is very close to zero for wavelengths shorter than $\lambda = 1$ μm, we focus the rest of our analysis on

the wavelength range from λ = 1-5 μm, where there is significant detectable radiation from our active device. Figure S20b plots the simulated reflectance from our device across this extended wavelength range for three carrier concentrations of the ITO depletion layer. (All assumptions in this simulation are identical to those used in the main text.) Here, we can see three distinct resonances. The designed resonance around λ = 4.9 μm corresponds to the magnetic dipole gap plasmon mode and is strongly modulated by the carrier concentration modulation. The resonance around λ = 1.9 μm corresponds to the electric dipole gap plasmon mode and is only slightly tuned by the carrier depletion. Finally, the narrow resonance around λ = 1.4 μm corresponds to the Rayleigh anomaly caused by the wavelength of light at normal incidence being equal to the antenna periodicity and is not significantly modulated. Next, we can calculate the expected radiance modulation from our device by first calculating the simulated emissivity. Because there is no transmission in our device, we can assume that the absorption is given by 1 – Reflectance. Then, by Kirchoff's law of thermal radiation, we know that the absorptivity is equal to the emissivity. Thus, we plot the expected spectral radiance from our device in Fig. S20c by multiplying the spectral radiance in Fig. S20a by (1 – R) from Fig. S20b at each carrier concentration. In this plot, we can more clearly see that the measured radiance is dominated by emission at wavelengths longer than λ = 3 μm. Additionally, there is slight modulation of the radiance at each simulated carrier concentration, which is strongest from wavelengths longer than λ = 4 μm. Finally, in Fig. S20d, we plot the absolute change in radiance between the maximum and minimum carrier concentrations from Fig. S20c. In this plot, we see that the largest change in measured radiance occurs at λ = 4.5 μm and significantly drops off for all other wavelengths. In conclusion, the active radiance modulation which we observe in the main text is a result of the modulation of the designed magnetic dipole gap plasmon mode, as expected.

Supplementary Figures and Tables

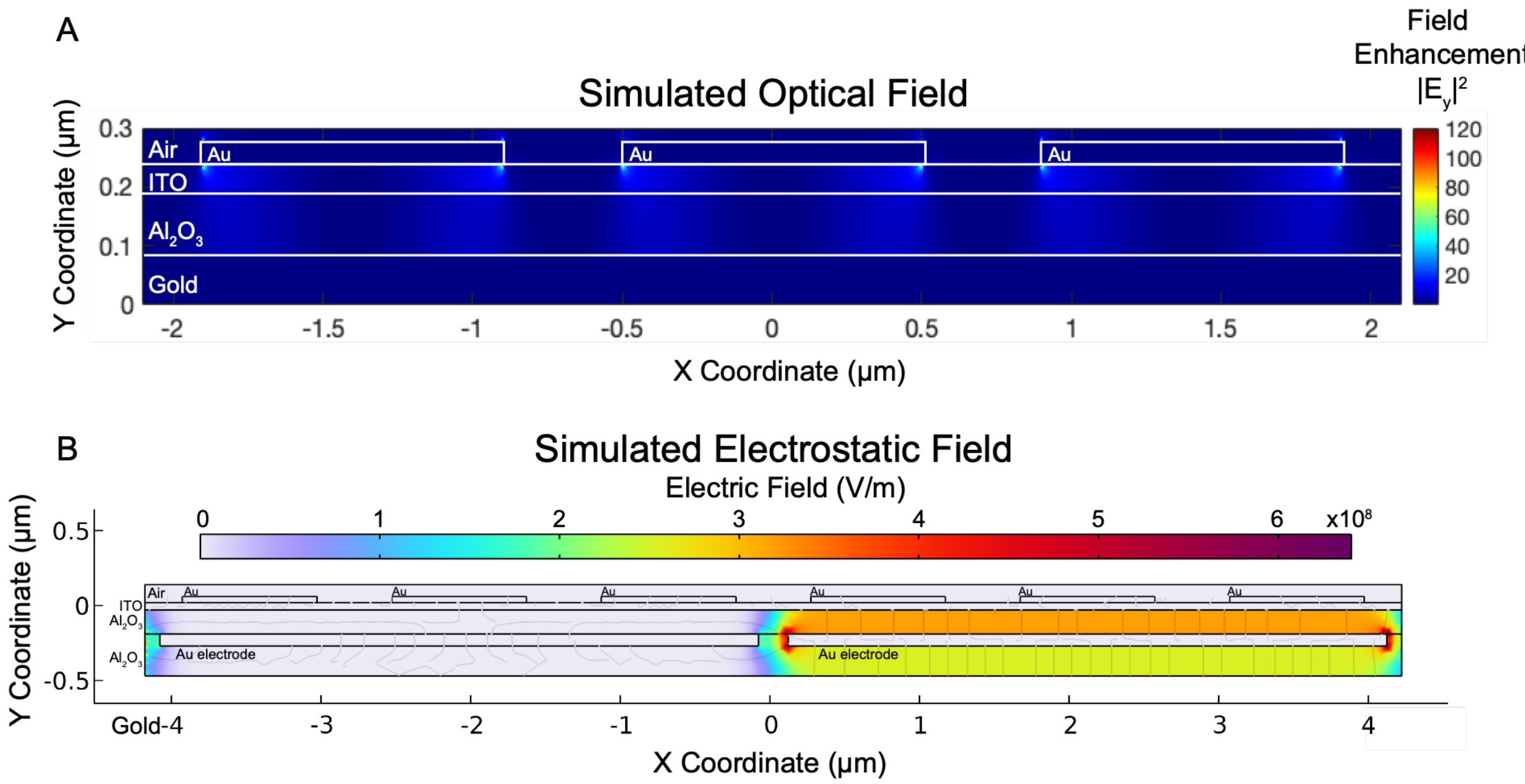

Figure S1: Simulated optical and electrical crosstalk. (A) Simulated y-component of optical electric field profile for a group of three antenna elements. **(B)** Simulated electrostatic field between two electrode groups (three antennas each) for an applied voltage of 0 V and –51 V between the continuous ITO layer and the left and right Au electrodes, respectively. Note: (A) plots a region consisting of three antennas which is half the size of (B), consisting of six antennas.

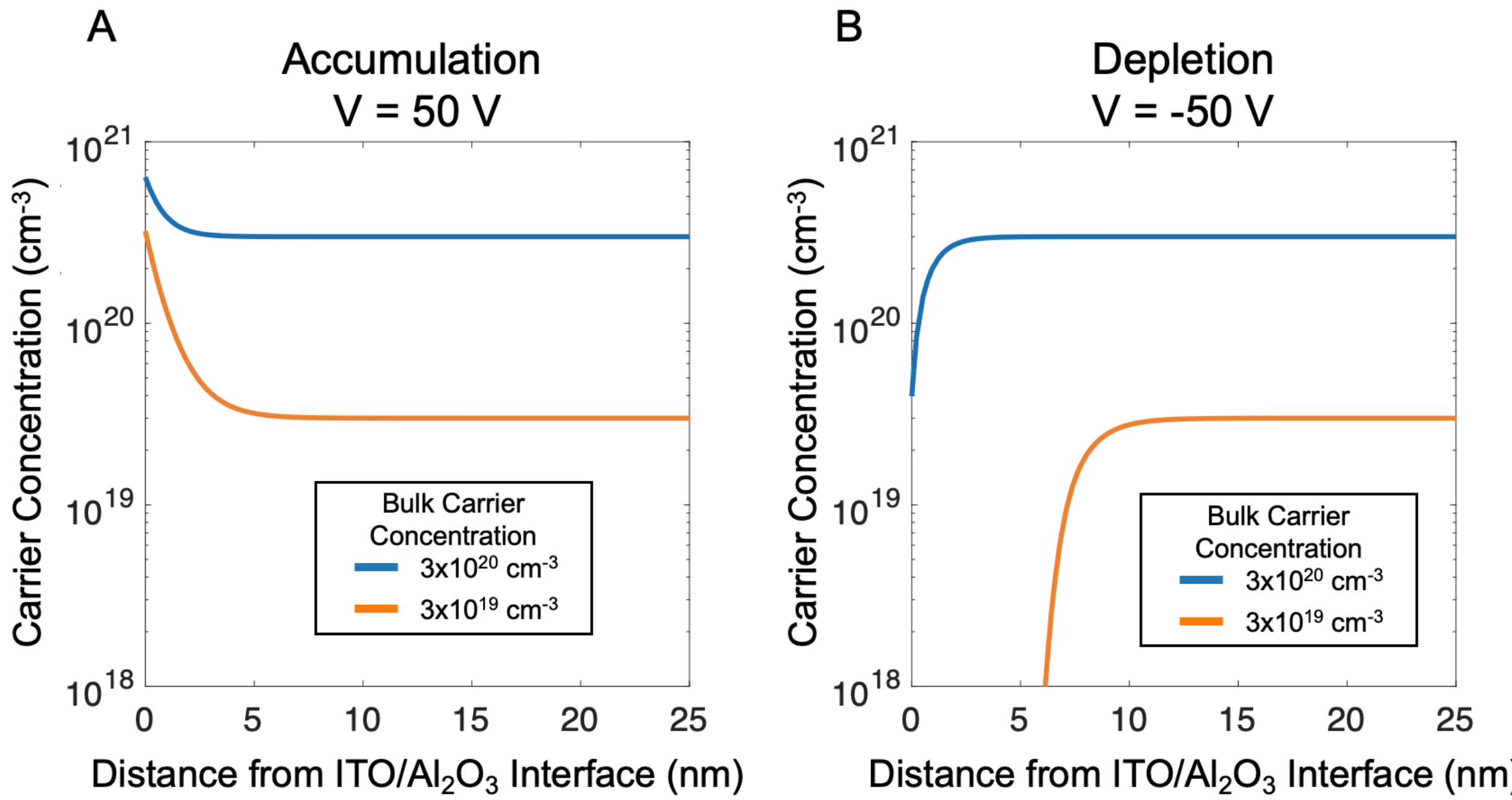

Figure S2: Electron density distribution in ITO films of different bulk carrier concentration. The simulated carrier concentration profile within 50 nm films of ITO with bulk carrier concentrations of $3x10^{20}$ cm^{-3} (blue line) and $3x10^{19}$ cm^{-3} (orange line). The carrier density is plotted as a function of the distance from the ITO/Al_2O_3 interface, for the first 25 nm of the ITO film, when a bias of 50 V **(A)** and -50 V **(B)** is applied.

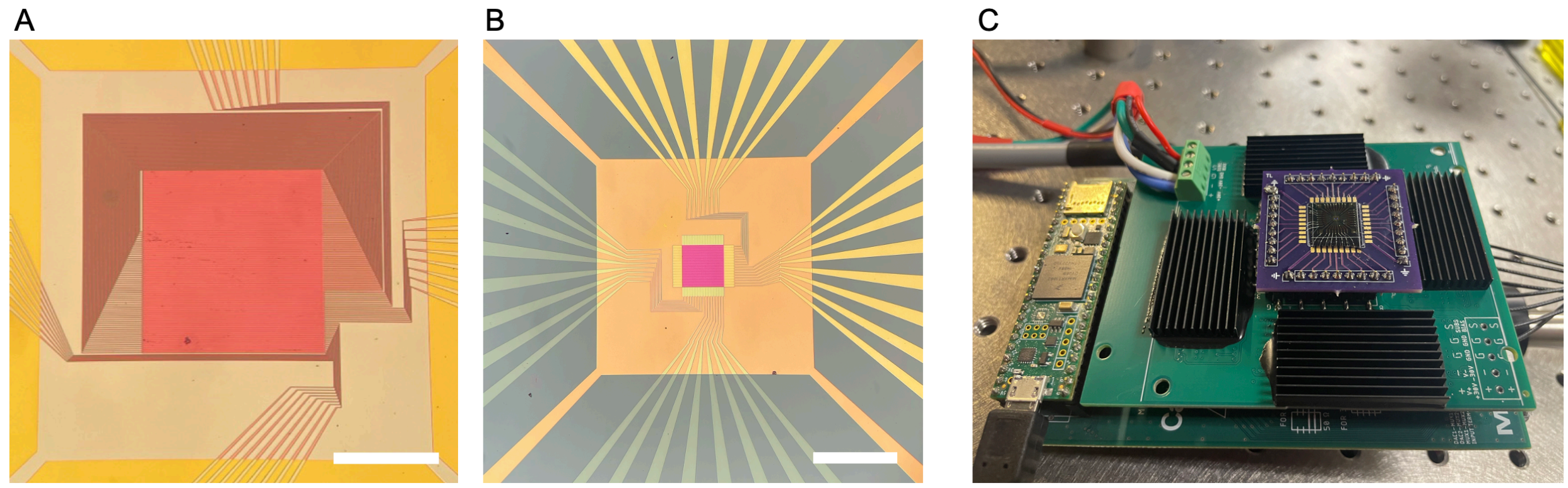

Figure S3: Optical images of active metasurface devices. (A) Optical microscope image of 1D device. Scale bar: 250 **μ**m. **(B)** Optical microscope image of 2D controllable device. Scale bar: 550 **μ**m. **(C)** Optical camera image of metasurface device connected to 32-channel driving PCB.

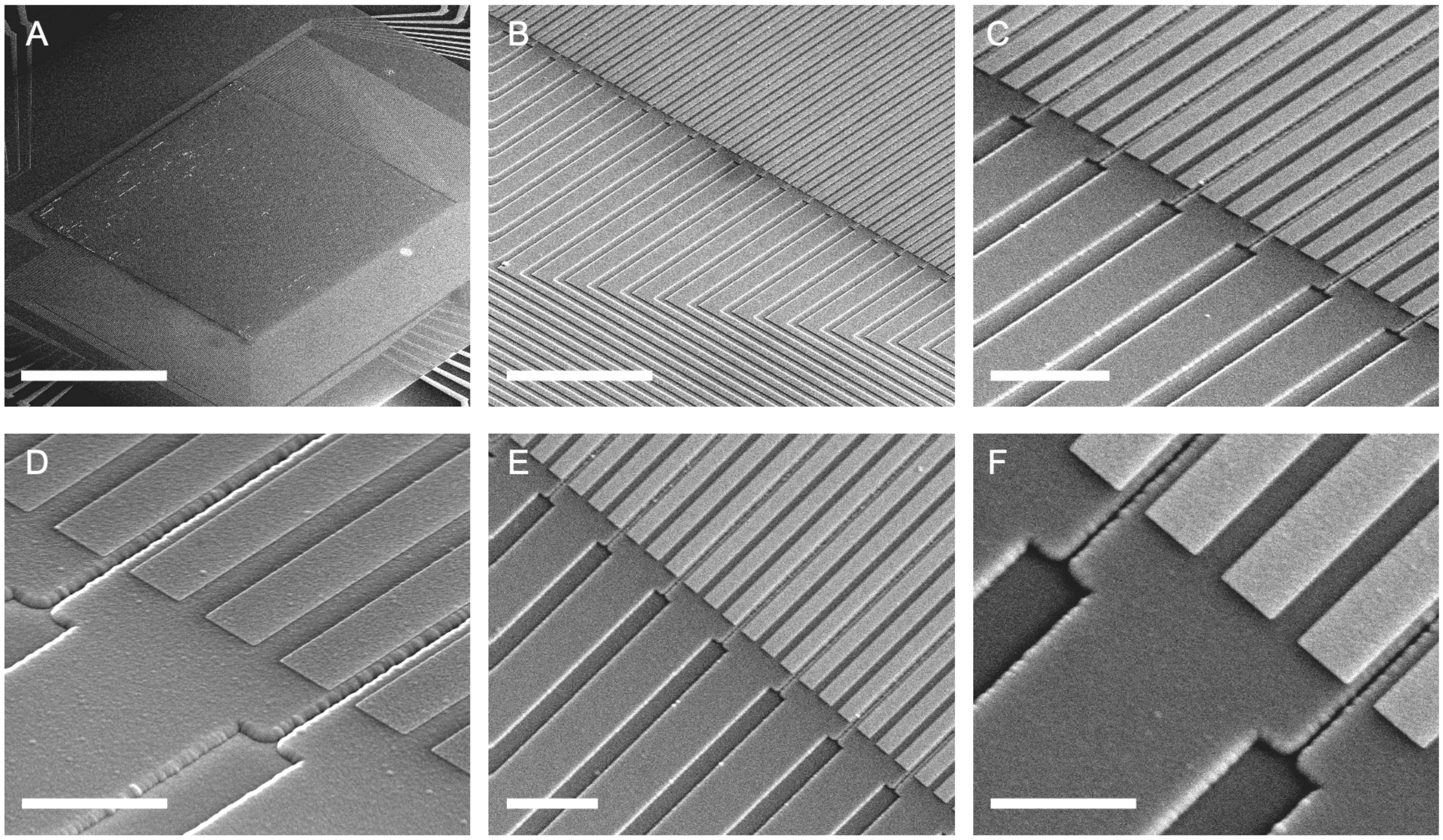

Figure S4: Scanning electron microscope (SEM) images of 1D structure. (A-D) Images taken at a tilt of 52°. **(E and F)** Images taken at normal incidence (no axis tilt). Scale bars for (A-F) are: 200 μm, 20 μm, 5 μm, 2 μm, 5 μm, and 2 μm, respectively.

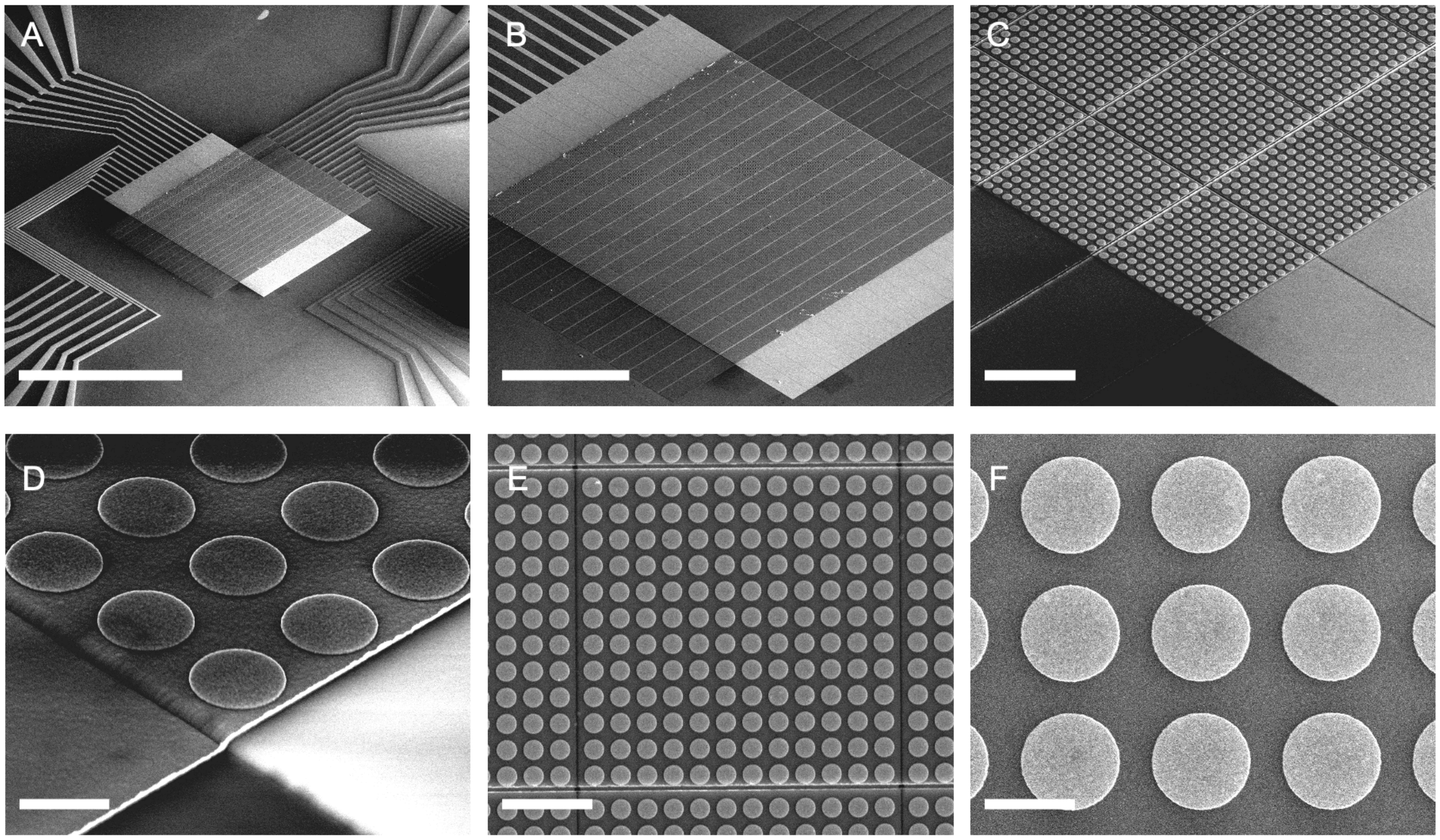

Figure S5: Scanning electron microscope (SEM) images of 2D structure. (A-D) Images taken at a tilt of 52°. **(E and F)** Images taken at normal incidence (no axis tilt). Scale bars for (A-F) are: 300 µm, 100 µm, 10 µm, 1 µm, 5 µm, and 1 µm, respectively.

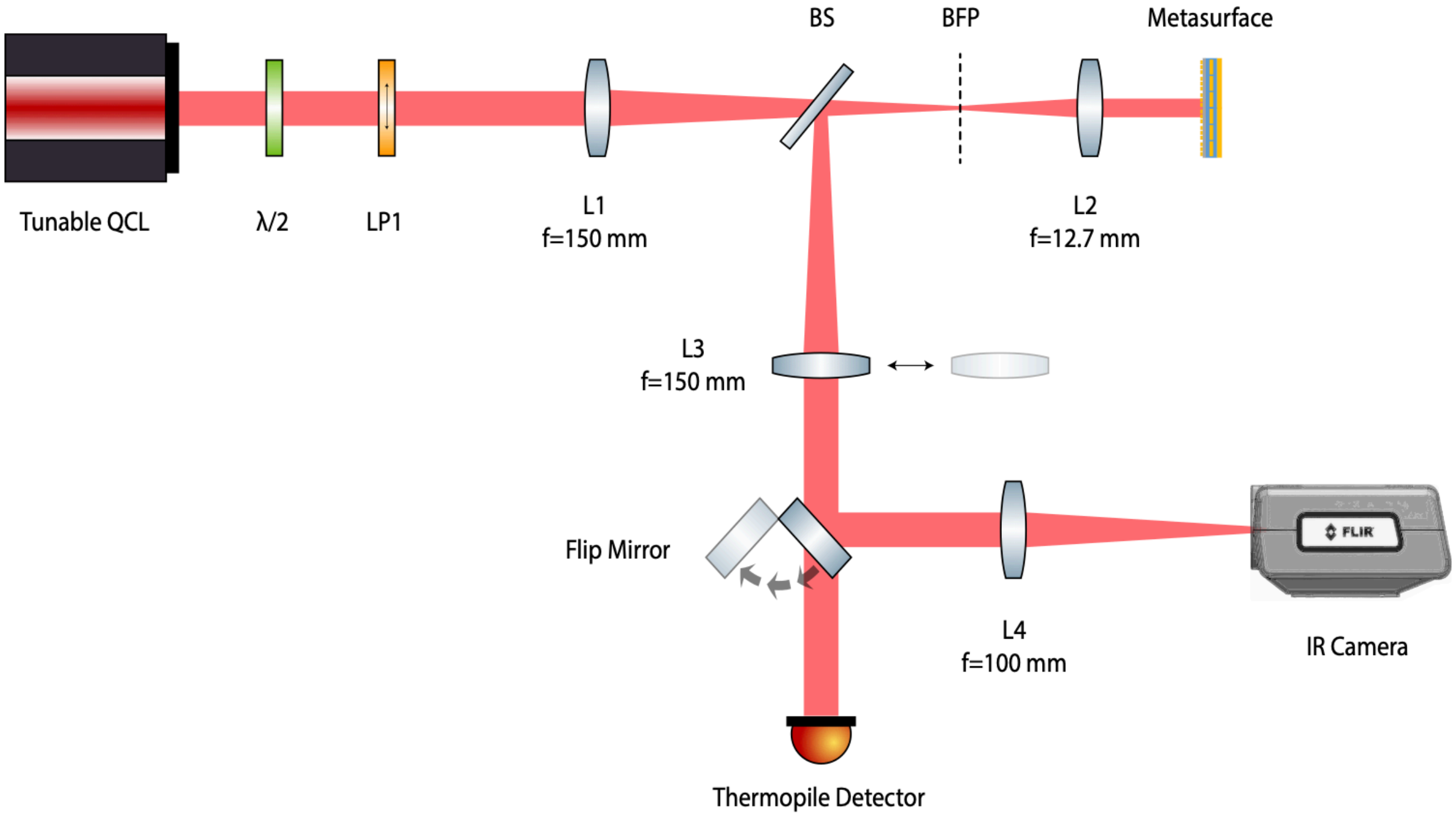

Figure S6: Experimental setup for active metasurface characterization.

Table S1: Comparison of mid-IR beam steering platforms. Dashes indicate the metric is not applicable; N/R indicates the metric was not reported.

Platform	Total Efficiency (%)	Linear Resolvable Spots	Switching Speed	λ (μm)	Static Power	Ref.
Non-mechanical						
This work	0.3	80 (32 usable)	> 150 kHz	4.95	0.6 mW	
OPA (1D)	1.5	38	∼ 1 kHz	4.6	225 mW	*(11)*
OPA (2D)	0.32	35	N/R	4.6	1.35 W	*(49)*
OPA (1D)	N/R	14	N/R	3.5	104 mW	*(55)*
LC SEEOR	3	24	N/R	4.6	500 V drive	*(48)*
AOD + nonlinear crystal	2.8	50	∼ 1 MHz	3.47	2 W (AOD)	*(50)*
Diffractive LC waveplate	98	2	∼ 1 Hz	4.5	> 80 V	*(5)*
AOD (Brimrose)	> 40	200	50 MHz	2.1	> 1 W	*(51)*
AOD (Isomet)	80	436	91 kHz	9.3	> 100 W	*(52)*
AOD (Intra Action)	80	20	1 MHz*	2.5–11	30 W	*(53)*
Mechanical						
FSM (Optotune)	> 98	—	300 Hz	Broadband	1.5 W	*(54)*
Risley prism	> 99	—	50 Hz	2–5	N/R	*(46, 47)*

* Estimated assuming a 5.5 mm beam aperture

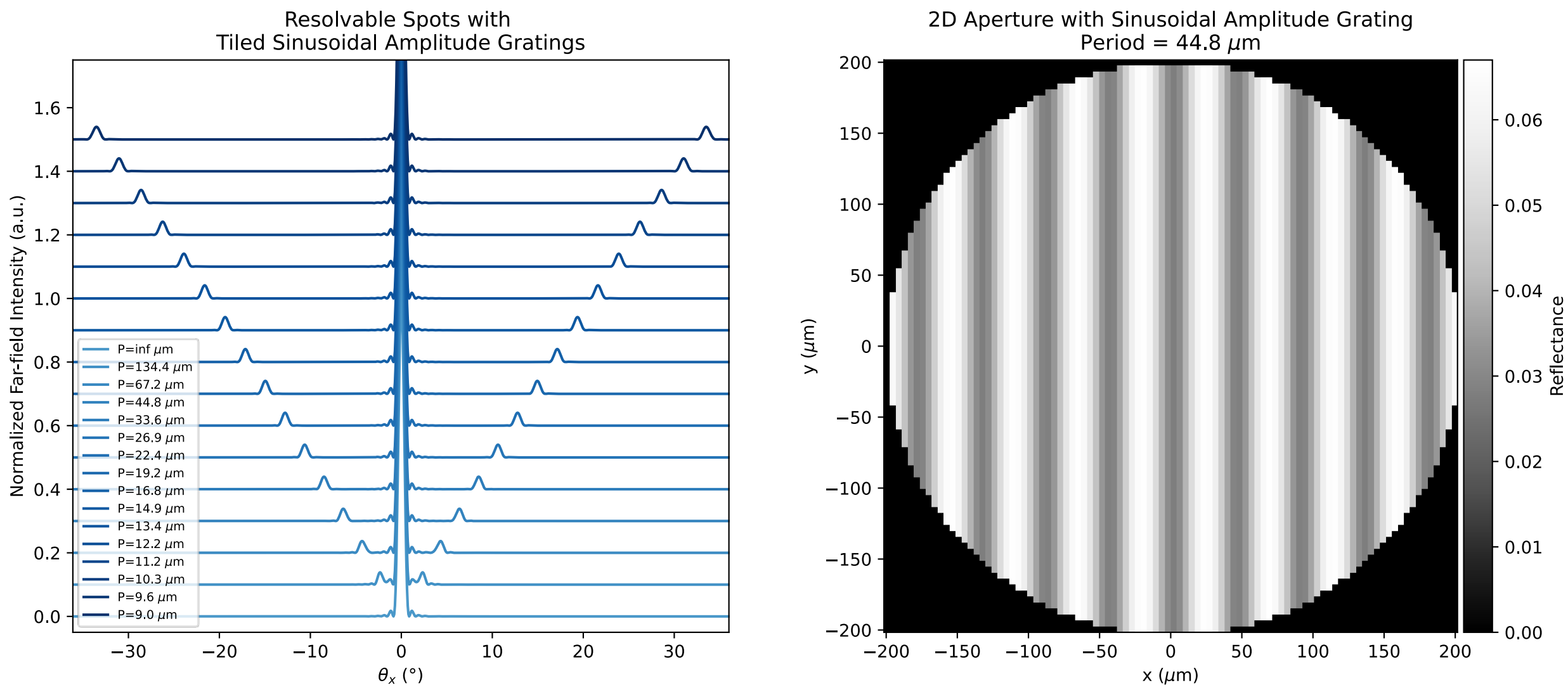

Figure S7: Simulated steering to all resolvable spots of the active metasurface. The left panel shows the simulated far-field intensity distribution for evenly spaced resolvable spots of the metasurface when sinusoidal amplitude grating patterns are applied across the tiled aperture. The legend indicates the period of the patterns. The right panel shows an example of the reflectance profile used to simulate the far-field intensity for a period of 44.8 μm.

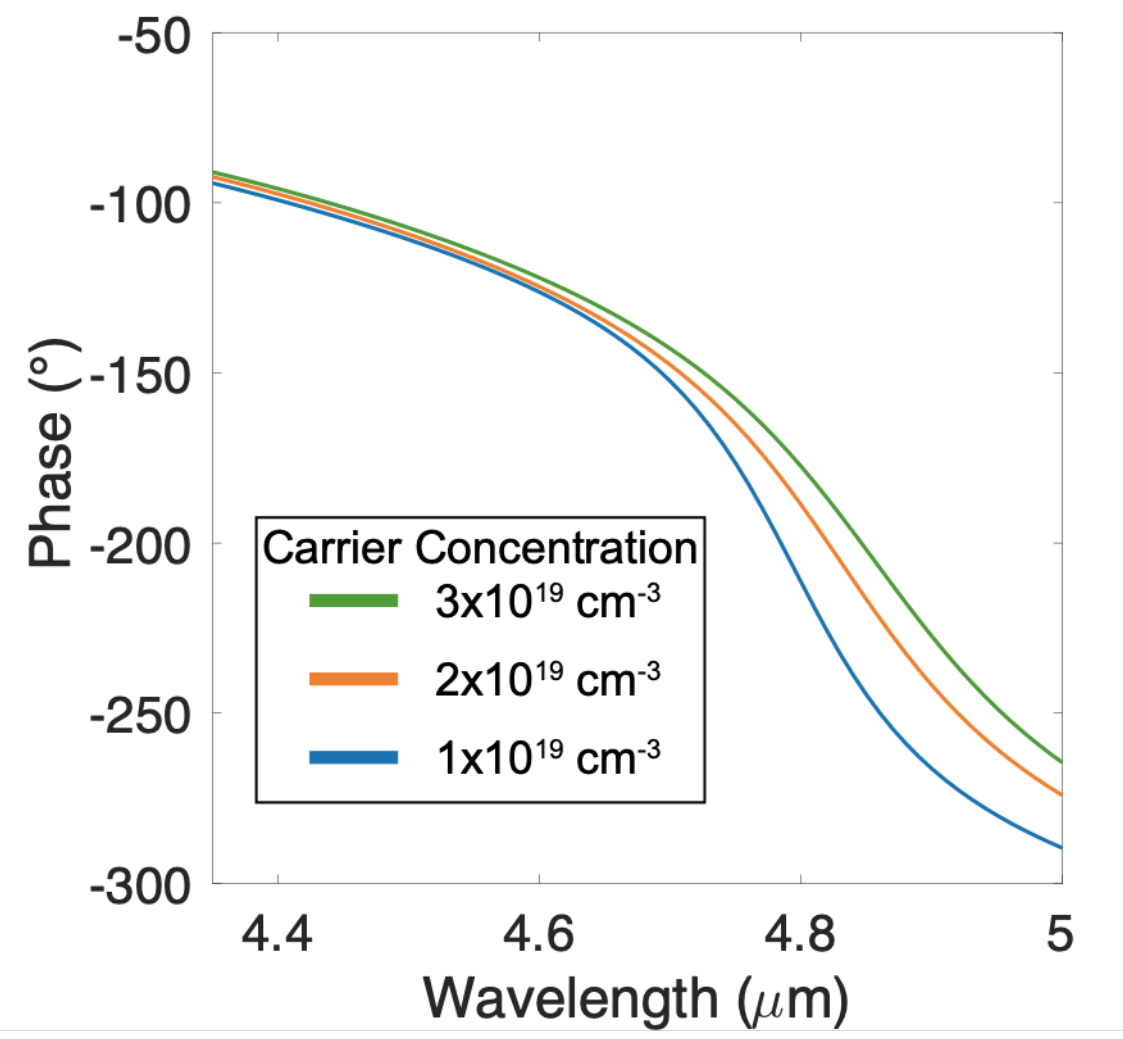

Figure S8: Simulated phase response of active metasurface. Simulated spectral phase curves as a function of assumed carrier concentration in a 10 nm depletion layer at the ITO/Al_2O_3 interface.

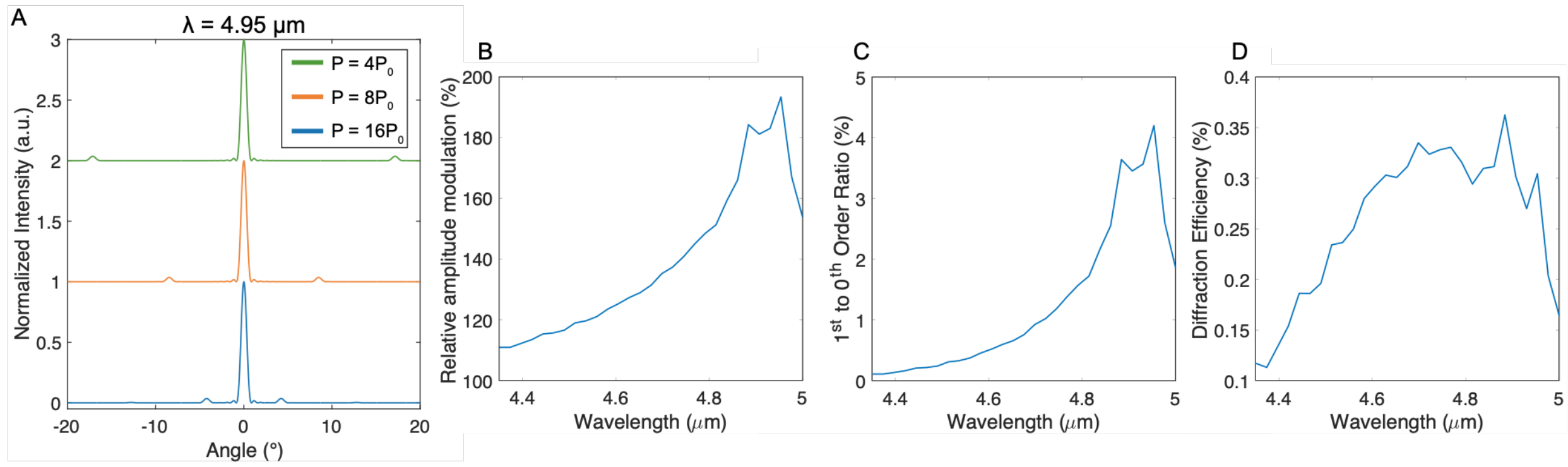

Figure S9: Array factor calculation of expected diffraction using experimentally measured reflectance modulation (Fig. 3d in main text). (A) Calculated far-field intensity distribution for three different binary diffraction grating periodicities for $\lambda = 4.95$ µm. $P_0 = 1.2$ µm. **(B)** Measured maximum relative amplitude modulation as a function of wavelength. Each value corresponds to the amplitude modulation between applied voltages of 0 V and –51 V. **(C)** Ratio of peak intensity in 1st order to 0th order spots from calculated far-field diffraction profiles in (A). **(D)** Total diffraction efficiency of power sent to +/- 1st diffraction orders.

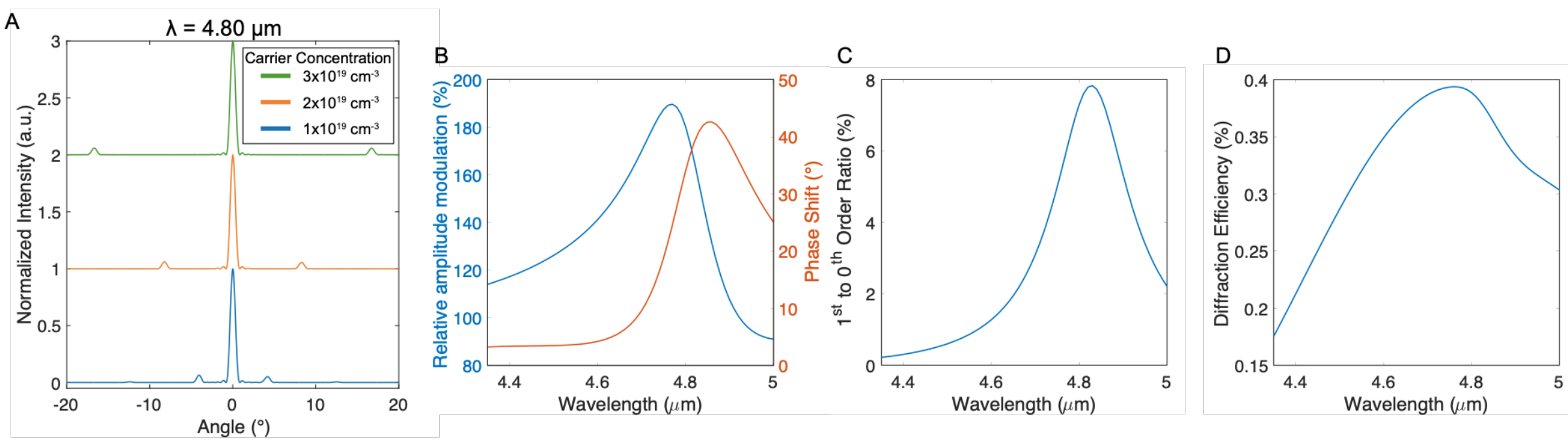

Figure S10: Array factor calculation of expected diffraction using simulated reflectance and phase modulation (Figs. 3c in main text and S8 in Supplementary Materials). (A) Calculated far-field intensity distribution for three different binary diffraction grating periodicities for λ = 4.80 µm. P_0 = 1.2 µm. **(B)** Maximum simulated relative amplitude and phase modulation as a function of wavelength. **(C)** Ratio of peak intensities in 1st and 0th orders from calculated far-field diffraction profiles in (A). **(D)** Total diffraction efficiency of power sent to +/- 1st diffraction orders.

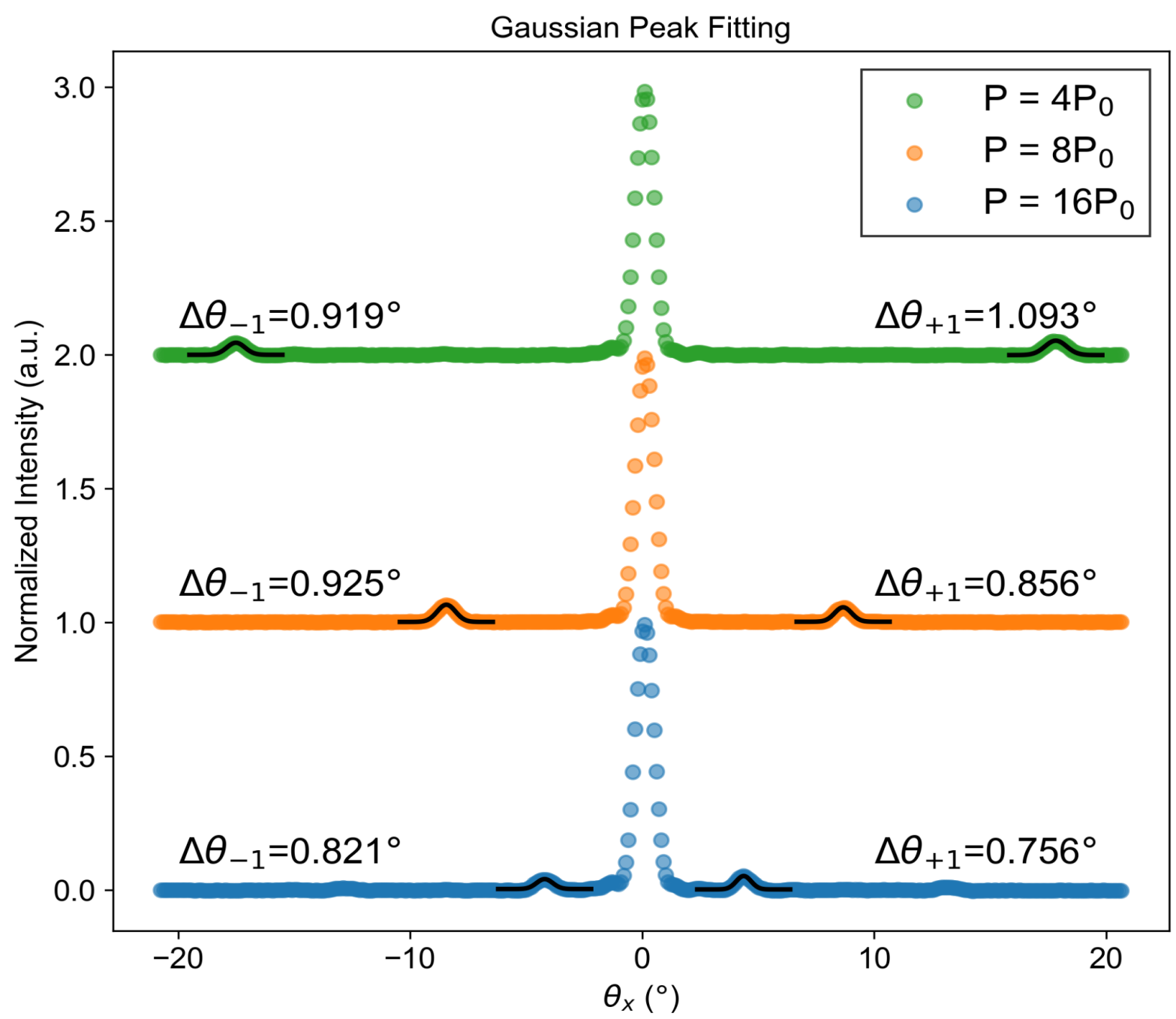

Figure S11: Analysis of diffraction order divergence. Fourier plane images (same as in Fig. 4c of the main text) with overlaid Gaussian fits for the diffraction orders and labeled FWHM for each order.

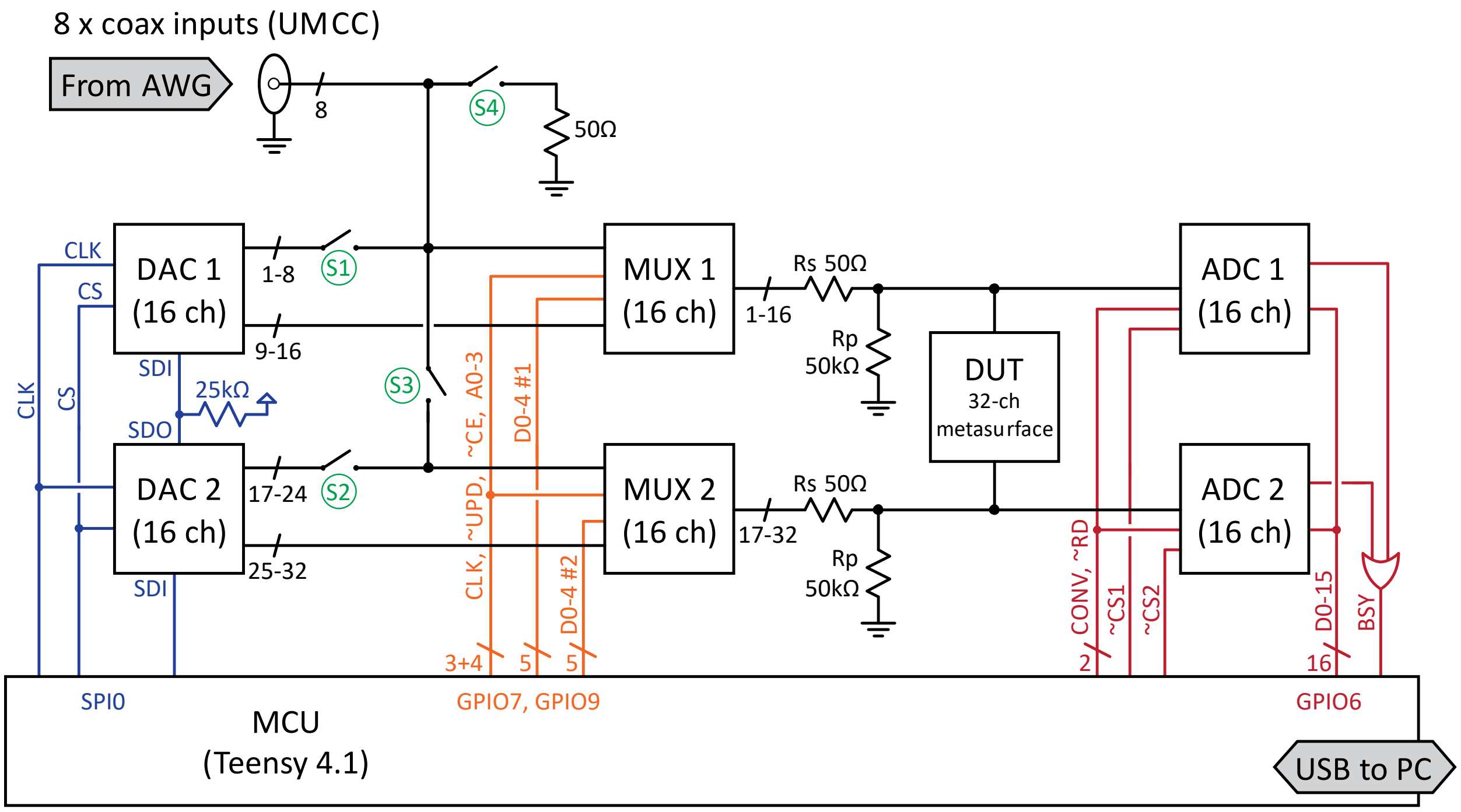

Figure S12: Simplified schematic of custom biasing circuitry. Switch banks S1 and S2 are closed to utilize the onboard DACs; S3 and S4 are closed to utilize external AWG inputs.

Table S2: Summary of biasing modes and approximate usable bandwidth/reconfiguration speeds. Values are observations, not necessarily the limiting performance of constituent components.

Dynamic Biasing Mode	Unique Channels	Analog BW	Reconfiguration Bandwidth
External AWG	8	3 MHz	N/A
MUX remapping (static voltages)	16	3 MHz	250 kS/s
DAC Toggling (two states)	32	80 kHz	250 kS/s
DAC AWG (∼4k states)	32	80 kHz	25 kS/s

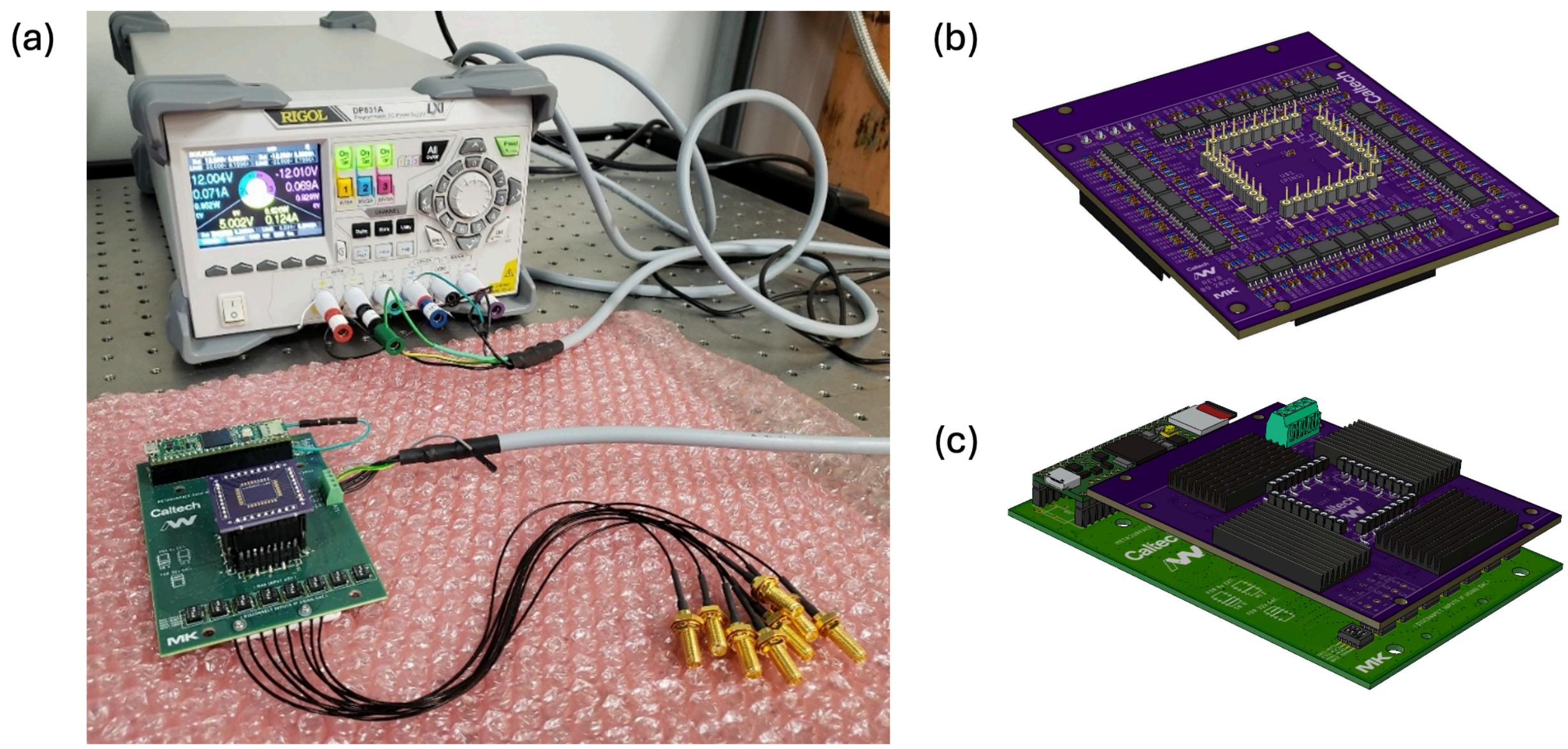

Figure S13. Driving electronics. (A) Photograph of original biasing PCB with unpopulated chip carrier in socket. **(B)**,**(C)** Renderings of amplifier interposer board to increase biasing range to ±30V.

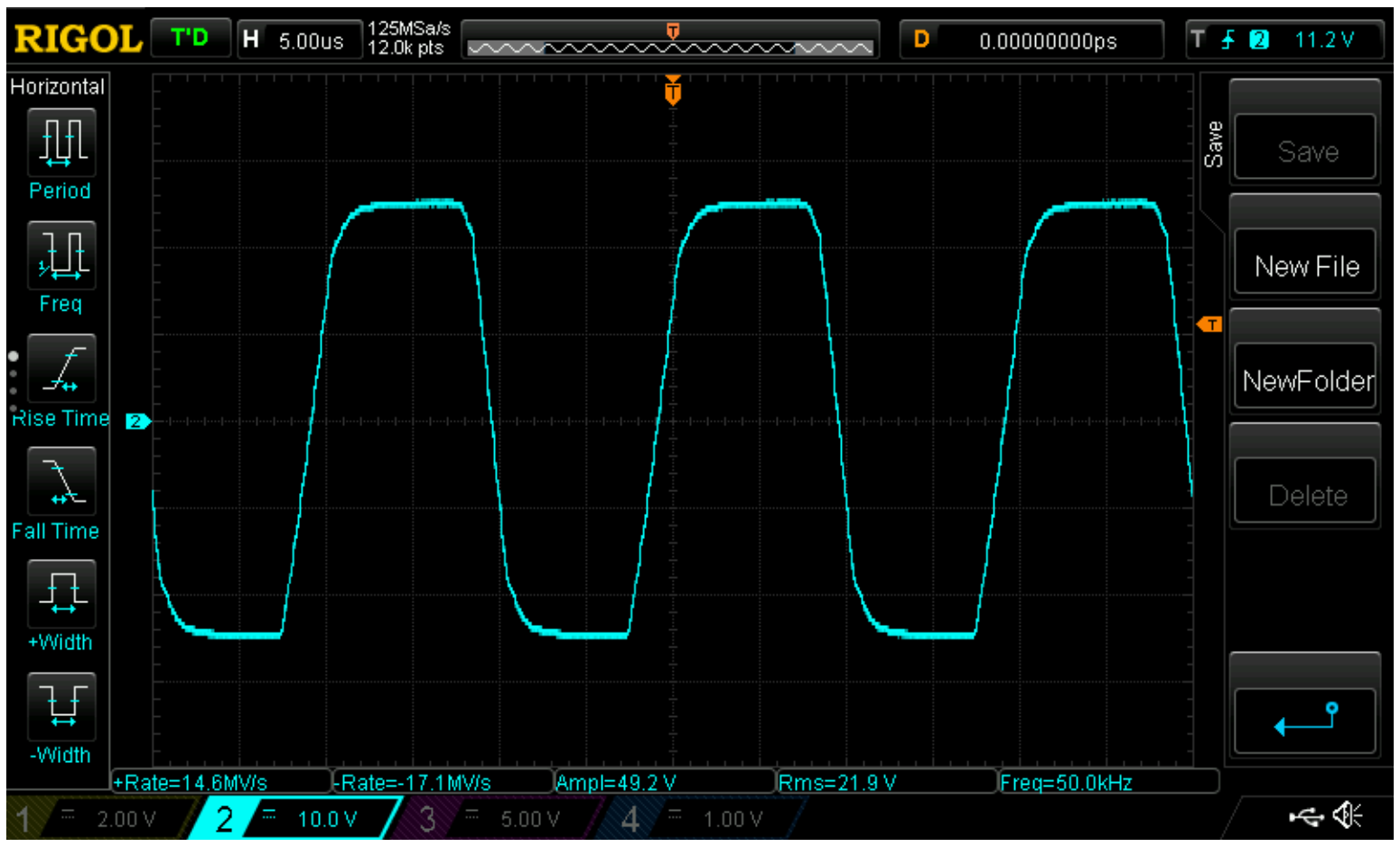

Figure S14. **Oscilloscope image showing typical output swing waveform from voltage amplifier board.** Signal is nominal 50 kHz, 50 $V_{p\text{-}p}$ square wave generated from onboard DAC. The output swing rate is limited by both the DAC and amplifier slew rates in this case.

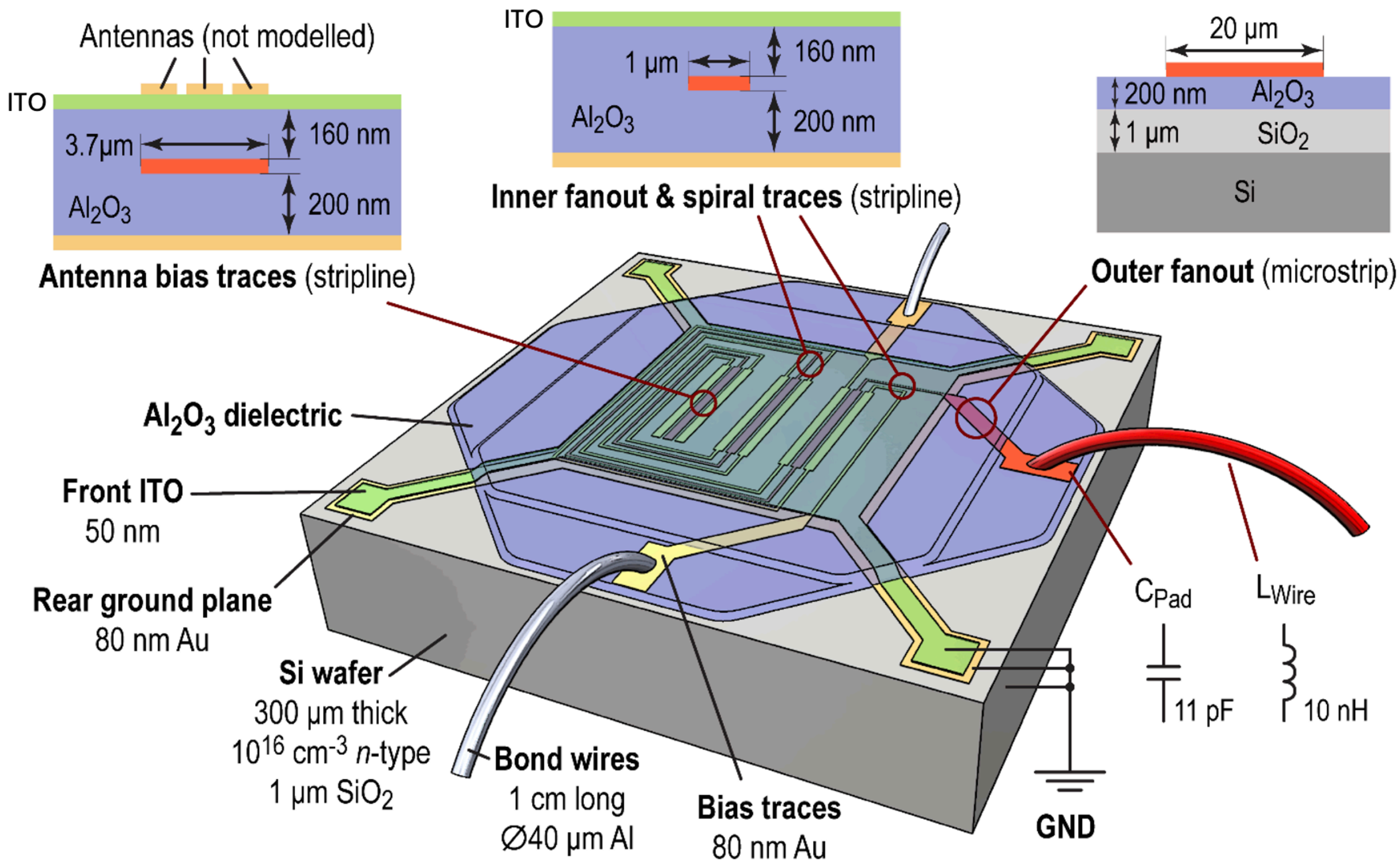

Figure S15. **Notional, simplified depiction of experimental device layout** (not to scale), showing key dimensions and properties used to produce a lumped-element circuit model. The illustration shows only (3) bias channels, actual devices had 32. The single channel modeled for simulation is highlighted in red. The Au antennas are omitted from the rendering, as they don't display clearly at this scale.

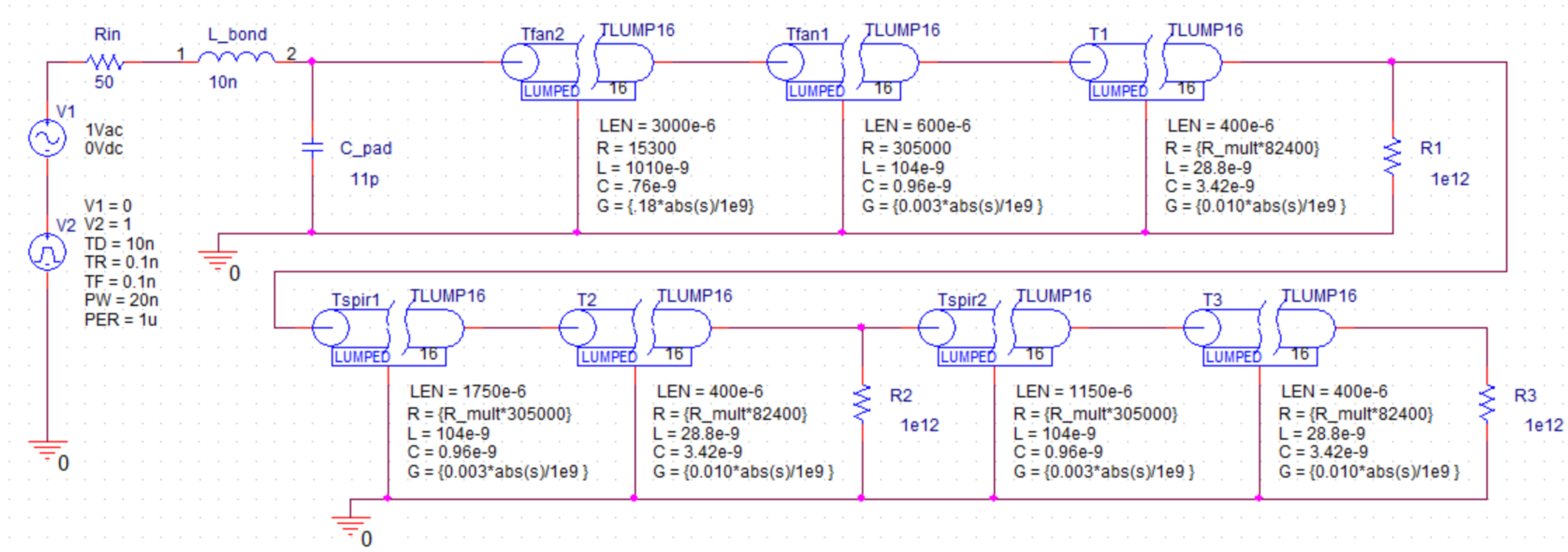

Figure S16. SPICE simulation circuit schematic for AC and transient analysis. (R1, R2, and R3 indicate the ends of each metasurface bias row and do not represent modeled elements.)

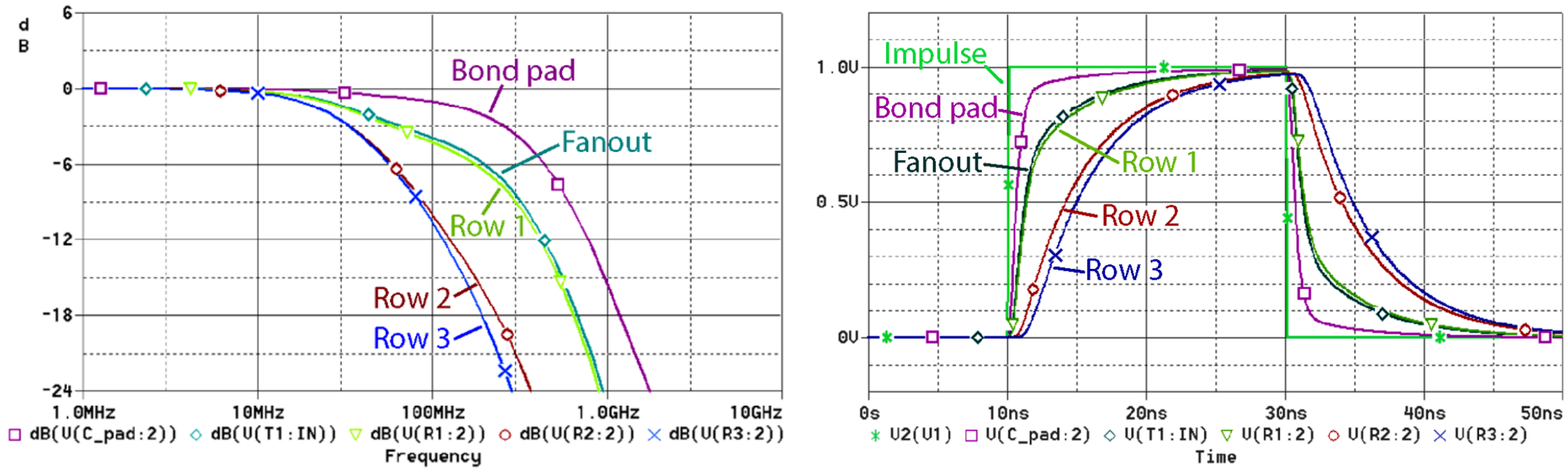

Figure S17. SPICE simulation results for AC (left) and 1V step transient (right) simulations.

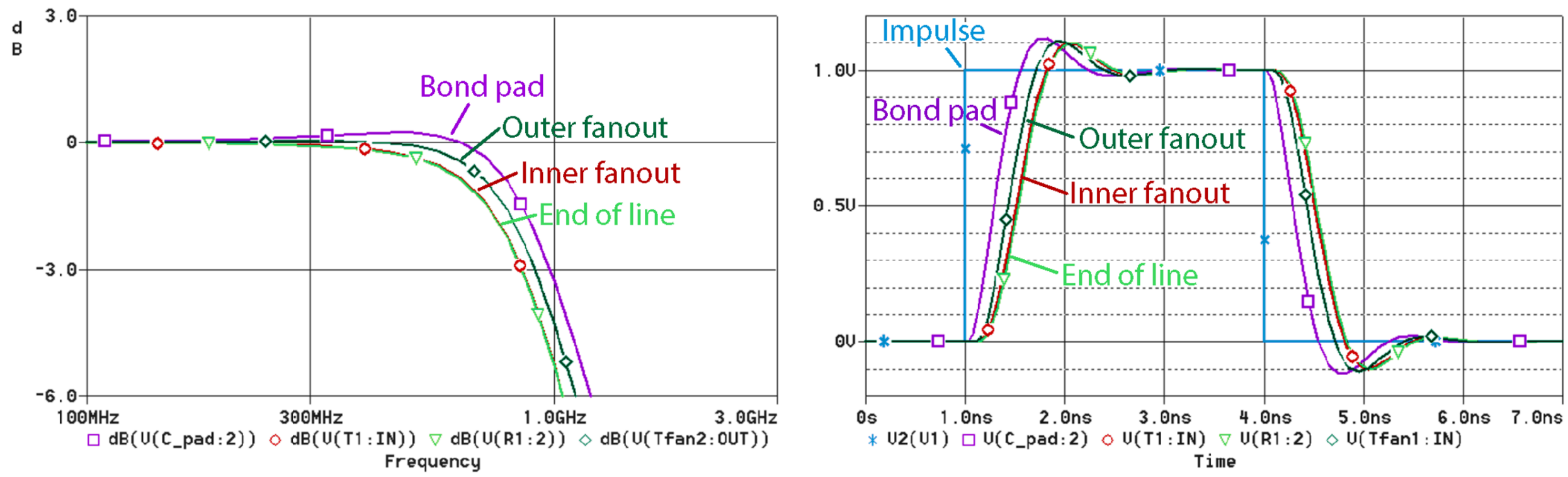

Figure S18. SPICE simulation results for AC (left) and 1V step transient (right), for the revised higher speed fanout device.

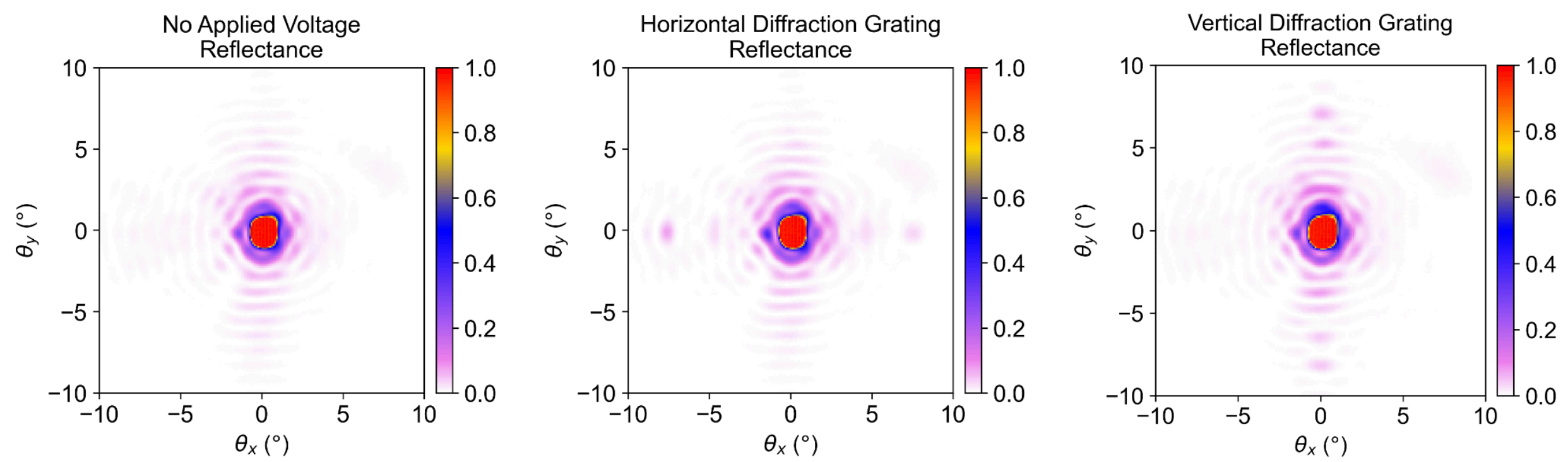

Figure S19: Measured Fourier plane images with X-polarized illumination. This is the X-polarized equivalent to the diffraction results presented in Fig. 5 of the main text for the 2D active metasurface.

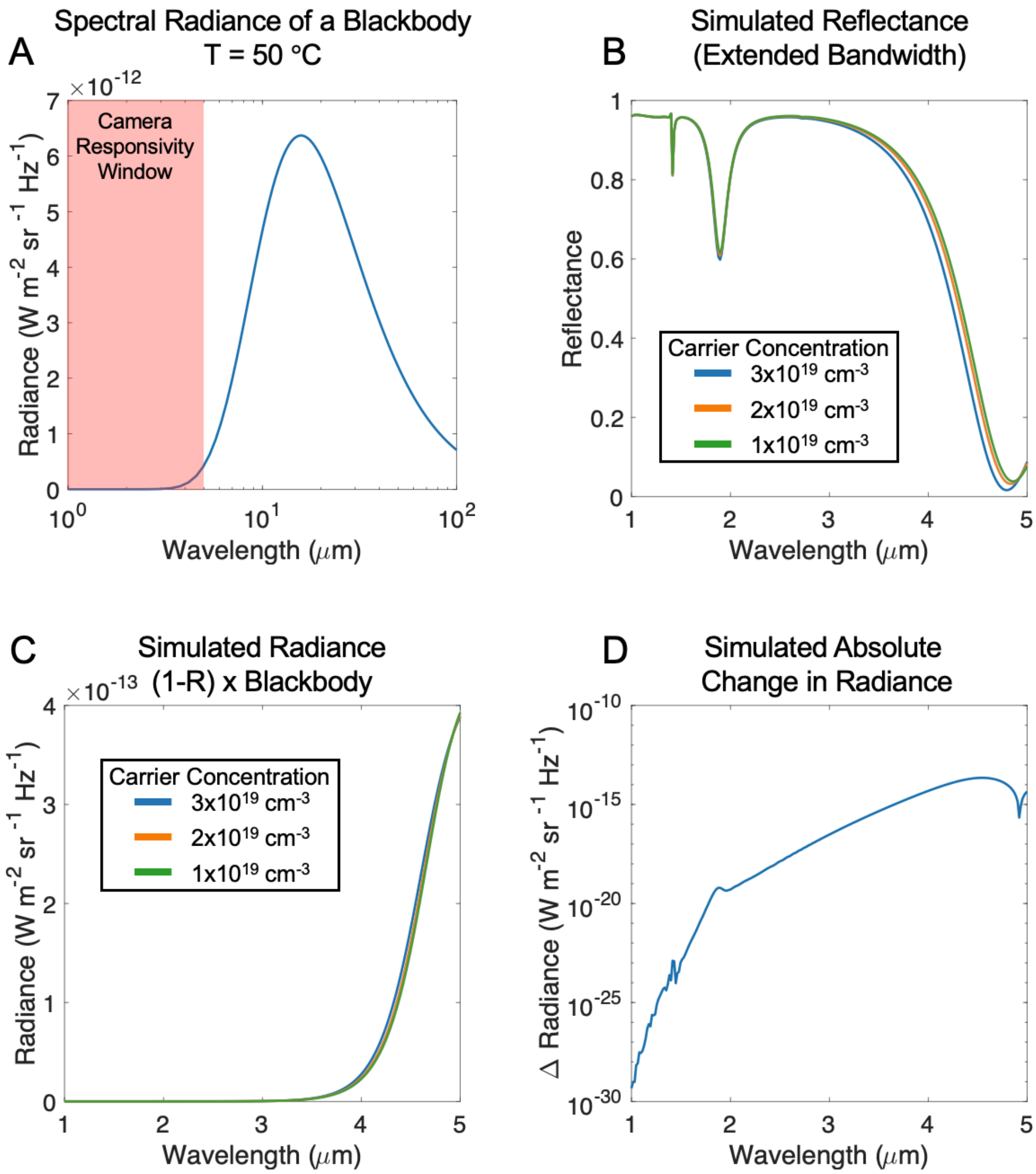

Figure S20: Spectral radiance analysis of our active metasurface. (A) Spectral radiance of a blackbody at T=50 °C calculated using Planck's Law. The responsivity window of the camera used in our measurements is shown in the red shaded region. **(B)** Simulated reflectance modulation of our active metasurface across a larger bandwidth for different assumed carrier concentrations in the ITO depletion layer. **(C)** Simulated total radiance modulation for our active metasurface. This plot was calculated by subtracting the simulated reflectance in (B) from 1 to obtain the absorption/emissivity, then multiplying by the radiance in (A). **(D)** Simulated absolute change in radiance in our active metasurface. This is obtained by subtracting the minimum radiance from the maximum radiance at each wavelength in (C).

Supplementary Video S1: Repeated active diffractive switching to 3 different angles, holding for 0.5 seconds at each angle. This corresponds to the 3 diffraction patterns plotted in Figs. 4b,c in the main text.

Supplementary Video S2: Imaging of thermal radiation modulation while applying voltage in vertical strips to the device, holding for 0.5 seconds in each state. This corresponds to switching between the states plotted in Figs. 5f,g in the main text.

REFERENCES

1. J. Meng, S. Balendhran, Y. Sabri, S. K. Bhargava, K. B. Crozier, Smart mid-infrared metasurface microspectrometer gas sensing system. *Microsyst. Nanoeng.* **10**, 74 (2024).

2. S. J. Byrnes, R. Blanchard, F. Capasso, Harvesting renewable energy from Earth's mid-infrared emissions. *Proc. Natl. Acad. Sci. U.S.A.* **111**, 3927–3932 (2014).

3. A. Khoshakhlagh, S. D. Gunapala, Infrared imaging: A potential powerful tool for neuroimaging and neurodiagnostics. *Neurophotonics* **4**, 011014 (2017).

4. F. Bao, X. Wang, S. H. Sureshbabu, G. Sreekumar, L. Yang, V. Aggarwal, V. N. Boddeti, Z. Jacob, Heat-assisted detection and ranging. *Nature* **619**, 743–748 (2023).

5. F. Gou, F. Peng, Q. Ru, Y.-H. Lee, H. Chen, Z. He, T. Zhan, K. L. Vodopyanov, S.-T. Wu, Mid-wave infrared beam steering based on high-efficiency liquid crystal diffractive waveplates. *Opt. Express* **25**, 22404–22410 (2017).

6. D. B. Beasley, M. Bender, J. Crosby, T. Messer, "Dynamic infrared scene projectors based upon the DMD," in *Proceedings of the SPIE*, L. J. Hornbeck, M. R. Douglass, Eds. (SPIE, 2009), p. 72100I.

7. P. Gattinger, I. Zorin, A. Ebner, C. Rankl, M. Brandstetter, Mid-infrared DMD-based spectral-coding spectroscopy with a supercontinuum laser source. *Opt. Express* **30**, 6440–6449 (2022).

8. M. Rutkauskas, A. Srivastava, D. T. Reid, "Spectral shaping of mid-infrared laser pulses with a digital micromirror device," in *Conference on Lasers and Electro-Optics* (*CLEO*) (IEEE, 2020), pp. 1–2.

9. G. Gauthier, I. Lenton, N. McKay Parry, M. Baker, M. J. Davis, H. Rubinsztein-Dunlop, T. W. Neely, Direct imaging of a digital-micromirror device for configurable microscopic optical potentials. *Optica* **3**, 1136–1143 (2016).

10. N. T. Ersumo, C. Yalcin, N. Antipa, N. Pégard, L. Waller, D. Lopez, R. Muller, A micromirror array with annular partitioning for high-speed random-access axial focusing. *Light Sci. Appl.* **9**, 183 (2020).

11. J. Midkiff, K. M. Yoo, J.-D. Shin, H. Dalir, M. Teimourpour, R. T. Chen, Optical phased array beam steering in the mid-infrared on an InP-based platform. *Optica* **7**, 1544–1547 (2020).

12. M. U. Pralle, N. Moelders, M. P. McNeal, I. Puscasu, A. C. Greenwald, J. T. Daly, E. A. Johnson, T. George, D. S. Choi, I. El-Kady, R. Biswas, Photonic crystal enhanced narrow-band infrared emitters. *Appl. Phys. Lett.* **81**, 4685–4687 (2002).

13. J. A. Mason, S. Smith, D. Wasserman, Strong absorption and selective thermal emission from a midinfrared metamaterial. *Appl. Phys. Lett.* **98**, 241105 (2011).

14. T. Inoue, M. De Zoysa, T. Asano, S. Noda, Realization of narrowband thermal emission with optical nanostructures. *Optica* **2**, 27 (2015).

15. J.-J. Greffet, R. Carminati, K. Joulain, J.-P. Mulet, S. Mainguy, Y. Chen, Coherent emission of light by thermal sources. *Nature* **416**, 61–64 (2002).

16. J. Yu, R. Qin, Y. Ying, M. Qiu, Q. Li, Asymmetric directional control of thermal emission. *Adv. Mater.* **35**, e2302478 (2023).

17. J. R. Nolen, A. C. Overvig, M. Cotrufo, A. Alù, Local control of polarization and geometric phase in thermal metasurfaces. *Nat. Nanotechnol.* **19**, 1627–1634 (2024).

18. S. Vassant, I. Moldovan Doyen, F. Marquier, F. Pardo, U. Gennser, A. Cavanna, J. L. Pelouard, J. J. Greffet, Electrical modulation of emissivity. *Appl. Phys. Lett.* **102**, 0871125 (2013).

19. V. W. Brar, M. C. Sherrott, M. S. Jang, S. Kim, L. Kim, M. Choi, L. A. Sweatlock, H. A. Atwater, Electronic modulation of infrared radiation in graphene plasmonic resonators. *Nat. Commun.* **6**, 7032 (2015).

20. T. Inoue, M. De Zoysa, T. Asano, S. Noda, Realization of dynamic thermal emission control. *Nat. Mater.* **13**, 928–931 (2014).

21. J. Park, J.-H. Kang, X. Liu, S. J. Maddox, K. Tang, P. C. McIntyre, S. R. Bank, M. L. Brongersma, Dynamic thermal emission control with InAs-based plasmonic metasurfaces. *Sci. Adv.* **4**, eaat3163 (2018).

22. X. Liu, Y. Zhong, Z. Wang, T. Huang, S. Lin, J. Zou, H. Wang, Z. Wang, Z. Li, X. Luo, R. Cheng, J. Li, H. S. Yun, H. Wang, J. Kong, X. Zhang, S. Shen, Electrically programmable pixelated coherent mid-infrared thermal emission. *Nat. Commun.* **16**, 1665 (2025).

23. C.-C. Popescu, M. R. A. Peters, O. Maksimov, H. Bhandari, R. Sharma, K. A. Richardson, A. Majumdar, H. J. Kim, R. Chen, K. P. Dao, L. Ranno, B. Mills, D. Calahan, T. Gu, J. Hu, Two-dimensional pixel-level addressable mid-infrared metasurface spatial light modulator. *Nat. Commum.* **17**, 8393 (2026).

24. J. Siegel, S. Kim, M. Fortman, C. Wan, M. A. Kats, P. W. C. Hon, L. Sweatlock, M. S. Jang, V. W. Brar, Electrostatic steering of thermal emission with active metasurface control of delocalized modes. *Nat. Commun.* **15**, 3376 (2024).

25. O. Reshef, I. De Leon, M. Z. Alam, R. W. Boyd, Nonlinear optical effects in epsilon-near-zero media. *Nat. Rev. Mater.* **4**, 535–551 (2019).

26. M. Silveirinha, N. Engheta, Tunneling of electromagnetic energy through subwavelength channels and bends using ε-near-zero materials. *Phys. Rev. Lett.* **97**, 157403 (2006).

27. A. Alù, M. G. Silveirinha, A. Salandrino, N. Engheta, Epsilon-near-zero metamaterials and electromagnetic sources: Tailoring the radiation phase pattern. *Phys. Rev. B* **75**, 155410 (2007).

28. S. Molesky, C. J. Dewalt, Z. Jacob, High temperature epsilon-near-zero and epsilon-near-pole metamaterial emitters for thermophotovoltaics. *Opt. Express* **21**, A96 (2013).

29. M. I. Hossain, A. Salhi, A. Zekri, A. Abutaha, Y. Tong, S. Mansour, Studying room temperature RF magnetron-sputtered indium tin oxide (ITO) thin films for large scale applications. *Results Surf. Interfaces* **18**, 100383 (2025).

30. Z. Yu, I. R. Perera, T. Daeneke, S. Makuta, Y. Tachibana, J. J. Jasieniak, A. Mishra, P. Bäuerle, L. Spiccia, U. Bach, Indium tin oxide as a semiconductor material in efficient p-type dye-sensitized solar cells. *NPG Asia Mater.* **8**, e305 (2016).

31. C.-L. Tien, H.-Y. Lin, C.-K. Chang, C.-J. Tang, Effect of oxygen flow rate on the optical, electrical, and mechanical properties of DC sputtering ITO thin films. *Adv. Condens. Matter Phys.* **2018**, 2647282 (2018).

32. S. Song, T. Yang, J. Liu, Y. Xin, Y. Li, S. Han, Rapid thermal annealing of ITO films. *Appl. Surf. Sci.* **257**, 7061–7064 (2011).

33. Y. W. Huang, H. W. H. Lee, R. Sokhoyan, R. A. Pala, K. Thyagarajan, S. Han, D. P. Tsai, H. A. Atwater, Gate-tunable conducting oxide metasurfaces. *Nano Lett.* **16**, 5319–5325 (2016).

34. J. Park, B. G. Jeong, S. Il Kim, D. Lee, J. Kim, C. Shin, C. B. Lee, T. Otsuka, J. Kyoung, S. Kim, K.-Y. Yang, Y.-Y. Park, J. Lee, I. Hwang, J. Jang, S. H. Song, M. L. Brongersma, K. Ha, S.-W. Hwang, H. Choo, B. L. Choi, All-solid-state spatial light modulator with independent phase and amplitude control for three-dimensional LiDAR applications. *Nat. Nanotechnol.* **16**, 69–76 (2021).

35. G. K. Shirmanesh, R. Sokhoyan, P. C. Wu, H. A. Atwater, Electro-optically tunable multifunctional metasurfaces. *ACS Nano* **14**, 6912–6920 (2020).

36. L. A. Mayoral Astorga, M. Shabaninezhad, H. Northfield, S. Ntais, S. Rashid, E. Lisicka-Skrzek, H. Mehrvar, E. Bernier, D. Goodwill, L. Ramunno, P. Berini, Electrically tunable plasmonic metasurface as a matrix of nanoantennas. *Nanophotonics* **13**, 901–913 (2024).

37. Z. L. Miao, S. J. Chua, Y. W. Zhang, S. Tripathy, Transition of 3D to 2D growth modes of InAs grown on GaAs. *Appl. Surf. Sci.* **252**, 3436–3440 (2006).

38. S. Il Kim, J. Park, B. G. Jeong, D. Lee, K.-Y. Yang, Y.-Y. Park, K. Ha, H. Choo, Two-dimensional beam steering with tunable metasurface in infrared regime. *Nanophotonics* **11**, 2719–2726 (2022).

39. P. Thureja, R. Sokhoyan, C. U. Hail, J. Sisler, M. Foley, M. Y. Grajower, H. A. Atwater, Toward a universal metasurface for optical imaging, communication, and computation. *Nanophotonics* **11**, 3745–3768 (2022).

40. A. Davoyan, H. Atwater, Perimeter-control architecture for optical phased arrays and metasurfaces. *Phys. Rev. Appl.* **14**, 024038 (2020).

41. R. Sokhoyan, P. Thureja, J. Sisler, M. Grajower, K. Shayegan, E. Feigenbaum, S. Elhadj, H. A. Atwater, Electrically tunable conducting oxide metasurfaces for high power applications. *Nanophotonics* **12**, 239–253 (2023).

42. G. Kafaie Shirmanesh, R. Sokhoyan, R. A. Pala, H. A. Atwater, Dual-gated active metasurface at 1550 nm with wide (>300°) phase tunability. *Nano Lett.* **18**, 2957–2963 (2018).

43. Z. Fang, R. Chen, J. Zheng, A. I. Khan, K. M. Neilson, S. J. Geiger, D. M. Callahan, M. G. Moebius, A. Saxena, M. E. Chen, C. Rios, J. Hu, E. Pop, A. Majumdar, Ultra-low-energy programmable non-volatile silicon photonics based on phase-change materials with graphene heaters. *Nat. Nanotechnol.* **17**, 842–848 (2022).

44. S. Kim, S.-H. Lee, I. H. Jo, J. Seo, Y.-E. Yoo, J. H. Kim, Influence of growth temperature on dielectric strength of Al_2O_3 thin films prepared via atomic layer deposition at low temperature. *Sci. Rep.* **12**, 5124 (2022).

45. M. Shabaninezhad, L. Ramunno, P. Berini, Tunable plasmonics on epsilon-near-zero materials: The case for a quantum carrier model. *Opt. Express* **30**, 46501–46519 (2022).

46. A. J. Van Rynbach, N. Miller, G. Harlan, G. J. Phillips, J. Havran, D. Moore, J. B. Wamsley, C. S. Brust, "Risley prisms for broadband optical beam steering," in *Laser Radar Technology*

and Applications XXVIII, M. D. Turner, G. W. Kamerman, L. A. Magruder, Eds. (SPIE, 2023), pp. 75–80.

47. D. C. Weber, J. D. Trolinger, R. G. Nichols, A. K. Lal, "Diffractively corrected Risley prism for infrared imaging," in *Acquisition, Tracking, and Pointing XIV*, M. K. Masten, L. A. Stockum, Eds. (SPIE, 2000), p. 79.

48. J. A. Frantz, J. D. Myers, R. Y. Bekele, C. M. Spillmann, J. Naciri, J. Kolacz, H. G. Gotjen, V. Q. Nguyen, C. C. McClain, L. Brandon Shaw, J. S. Sanghera, Chip-based nonmechanical beam steerer in the midwave infrared. *J. Opt. Soc. Am. B* **35**, C29–C37 (2018).

49. J. Midkiff, P.-Y. Hsiao, P. T. Camp, R. T. Chen, Mid-infrared 2D nonredundant optical phased array of mirror emitters in an InGaAs/InP platform. *Opt. Express* **32**, 43329–43341 (2024).

50. J. Bourderionnet, A. Brignon, D. Dolfi, J.-P. Huignard, Mid-infrared laser beam steering based on Fourier transform OPO. *Adv. Opt. Technol.* **6**, 103–110 (2017).

51. Acousto-Optic Deflectors. www.brimrose.com/free-space-ao/.

52. D1011-G70L-10 High Power AO Deflector. https://isomet.com/defmidnir.html.

53. MODEL AGD-406B1 INFRARED ACOUSTO-OPTIC DEFLECTOR. https://intraaction.com/wp-content/uploads/2023/06/AGD-40B1.pdf.

54. Dual-axis fast steering mirror with position feedback MR-15-30. www.optotune.com/product/mr-15-30/.

55. M. Prost, Y.-C. Ling, S. Cakmakyapan, Y. Zhang, K. Zhang, J. Hu, Y. Zhang, S. J. Ben Yoo, Solid-state MWIR beam steering using optical phased array on germanium-silicon photonic platform. *IEEE Photonics J.* **11**, 1–9 (2019).
